# An Engineered Hierarchical Hydrogel with Immune Responsiveness and Targeted Mitochondrial Transfer to Augmented Bone Regeneration

**DOI:** 10.1002/advs.202406287

**Published:** 2024-09-11

**Authors:** Wenjin Cai, Shihua Mao, Ying Wang, Bicong Gao, Jiaying Zhao, Yongzheng Li, Yani Chen, Dong Zhang, Jintao Yang, Guoli Yang

**Affiliations:** ^1^ Stomatology Hospital School of Stomatology Zhejiang University School of Medicine Zhejiang Provincial Clinical Research Center for Oral Diseases Key Laboratory of Oral Biomedical Research of Zhejiang Province Cancer Center of Zhejiang University Engineering Research Center of Oral Biomaterials and Devices of Zhejiang Province Hangzhou 310000 P. R. China; ^2^ Zhejiang Key Laboratory of Plastic Modification and Processing Technology College of Materials Science & Engineering Zhejiang University of Technology Hangzhou 310014 P. R. China; ^3^ The Wallace H. Coulter Department of Biomedical Engineering Georgia Institute of Technology and Emory University Atlanta GA 30318 USA

**Keywords:** bioenergy metabolism, bone mesenchymal stem cells, bone regeneration, mitochondrial transfer

## Abstract

Coordinating the immune response and bioenergy metabolism in bone defect environments is essential for promoting bone regeneration. Mitochondria are important organelles that control internal balance and metabolism. Repairing dysfunctional mitochondria has been proposed as a therapeutic approach for disease intervention. Here, an engineered hierarchical hydrogel with immune responsiveness can adapt to the bone regeneration environment and mediate the targeted mitochondria transfer between cells. The continuous supply of mitochondria by macrophages can restore the mitochondrial bioenergy of bone marrow mesenchymal stem cells (BMSC). Fundamentally solving the problem of insufficient energy support of BMSCs caused by local inflammation during bone repair and regeneration. This discovery provides a new therapeutic strategy for promoting bone regeneration and repair, which has research value and practical application prospects in the treatment of various diseases caused by mitochondrial dysfunction.

## Graphical abstract

We have developed an engineered hierarchical hydrogel for immune‐responsive and mechanobiological, by incorporating zwitterionic polymerization to realize the connection between mechanical strength and targeted biological regulation. Rho GTPase 1 (Miro1) loaded macrophage‐targeted zwitterionic nanogels (MDV) regulated the mitochondrial homeostasis of BMSC through intercellular mitochondrial transfer, thereby promoting bone regeneration.

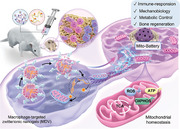

## 1 Introduction

Bone defects resulting from aging, severe trauma, and disease have perennially posed substantial clinical challenges, adversely impacting the functional and psychological well‐being of millions of patients.^[^ ^1^ ^]^ Conventional treatment strategies favor bone substitute (autografts, allografts, and xenografts) implantation for bone defect repair. Despite merits, potential complications related to immune response and local inflammation may cause treatment failure and worsen the condition.^[^ ^2^, ^3^ ^]^ The exploration of alternative therapeutic materials and mechanisms for bone defect therapy is necessitated by these constraints.

Bone defect repair initiates with inflammation, necessitating precise spatial and temporal control. In general, disruptions in this inflammatory cascade directly impede stem cell proliferation and differentiation, hindering subsequent repair progress.^[^ ^4^ ^]^ In response to bone injury, early responders in the form of congenital immune effector cells, specifically macrophages, come into play.^[^ ^5^ ^]^ The timely shift from pro‐inflammatory M1‐like macrophages to pro‐regenerative M2‐like macrophages represents a critical phase in normal bone regeneration.^[^ ^6^ ^]^ Research evidence has confirmed the ability of macrophages to regulate the osteogenic differentiation of bone marrow mesenchymal stem cells (BMSC).^[^ ^7^ ^]^ Upon the initiation of BMSC osteogenic differentiation, activated mitochondria generate a substantial amount of adenosine triphosphate (ATP) to facilitate cell mineralization.^[^ ^8^ ^]^ The study found that when affected by inflammatory factors, the level of ATP and oxidative phosphorylation (OXPHOS) decreased and the level of reactive oxygen species (ROS) increased in BMSC, which showed mitochondrial dysfunction. The metabolic remodeling of BMSC cells can lead to abnormal osteogenic differentiation.^[^ ^7^, ^9^ ^]^ However, bone regeneration materials that only regulate the polarity of macrophages do not fundamentally address the mitochondrial dysfunction and energy metabolism abnormalities of BMSC caused by inflammation.

Mitochondria, like “batteries,” are the essential energy sources of cells and regulate the functions of almost all cell types.^[^ ^10^ ^]^ Moreover, timely supplementation of healthy mitochondria is crucial for organs that require energy, such as the brain, muscles, and bones.^[^ ^11^ ^]^ Related studies have proposed using artificial supplementation of healthy mitochondria to maintain mitochondrial homeostasis as a repair method.^[^ ^12^ ^]^ However, reducing the damage to isolated mitochondria and maintaining their integrity are currently challenges for the application of this method.^[^ ^13^ ^]^ In recent years, intercellular mitochondrial transfer has received widespread attention as an alternative method.^[^ ^14^ ^]^ Recent studies have confirmed the critical role of mitochondrial transfer in tissue damage repair and homeostasis regulation.^[^ ^15^ ^]^ However, this transfer efficiency is insufficient to meet the demands of many mitochondrial transfers in the cell therapy industry.^[^ ^16^ ^]^ Previous studies have found that macrophages can regulate the energy metabolism and osteogenic function of BMSC through mitochondrial transfer.^[^ ^7a^ ^]^ Therefore, utilizing a safe and efficient mitochondrial transfer method to reconstruct the mitochondrial function of BMSC by “recharging” mitochondria may be a novel design strategy for bone regeneration materials.

**Figure 1:**
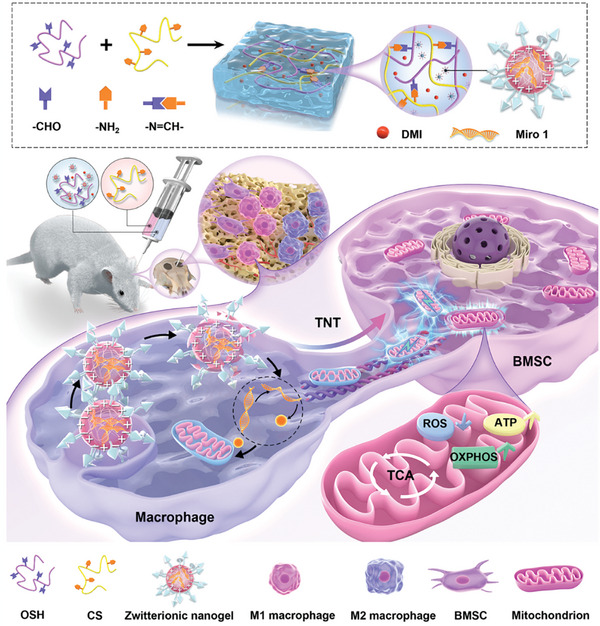
Schematic showing the engineered hierarchical hydrogel that realizes the targeted biological regulation. The engineered hierarchical hydrogel can provide a mechanical environment suitable for osteogenesis, and regulate the energy metabolism of BMSC by regulating inflammation and promoting intercellular mitochondrial transfer. Transferred mitochondria can enhance osteogenic differentiation and bone repair by reducing ROS levels in BMSC, and increasing ATP and OXPHOS levels.

Herein, we have created an engineered hierarchical hydrogel with immune response and targeted mitochondrial transfer, named Gel@MDI hydrogel, by utilizing reversible dynamic crosslinked networks and integrating both the anti‐inflammatory drug dimethyl itaconate (DMI) and macrophage‐targeted zwitterionic nanogels (MDV) (**Figure** **1**). Through reversible Schiff base bonding, the engineered hierarchical hydrogel demonstrated its ability to efficiently trigger the release of DMI in inflammatory settings, thereby facilitating the shift of macrophages toward the M2 phenotype. Notably, the MDV nanogels not only enhanced the mechanical properties of the hierarchical hydrogel for on‐site bone healing and regeneration but also exhibited exceptional efficiency in their role as gene carriers, resulting in superior gene transfection rates and reduced cytotoxicity.^[^ ^17^ ^]^ Therefore, Rho GTPase 1 (Miro1) loaded MDV nanogels with macrophage‐targeted function facilitated the mitochondrial transfer process from macrophages to BMSC. The “Mito‐Battery” can regulate the levels of ROS, ATP, and OXPHOS in BMSC through intercellular transfer, thereby affecting intracellular mitochondrial function and metabolic status and promoting the formation and growth of bone regeneration tissue. Our study elucidates the importance of intercellular mitochondrial transfer in bone defect repair and the role of immune and bioenergy‐regulated therapeutic strategies in tissue regeneration, providing new ideas for tissue regeneration engineering.

## 2 Results and Discussions

### 2.1 Tunneling Nanotubes Mediate Mitochondrial Transfer between Macrophages and BMSC

**Figure 2:**
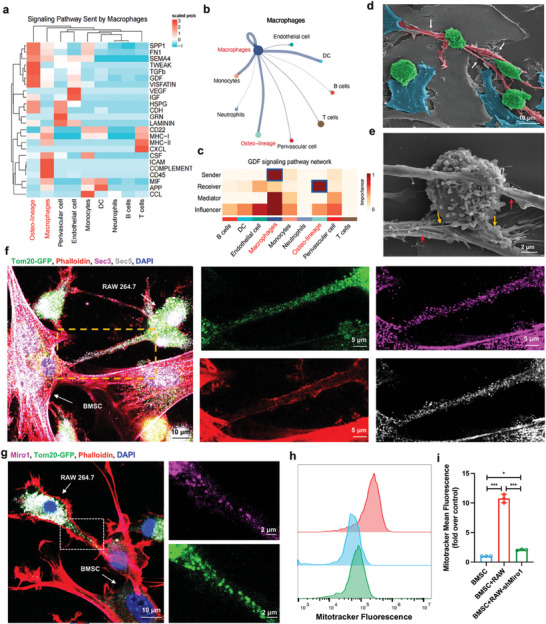
The connection between macrophages and BMSC via tunneling nanotubes. a) Heatmap illustrating the strength of signals emitted by macrophages. b) Circle plot showing a visual depiction of the interaction strength between macrophages and various other groups. c) Heatmap showing the functions of the GDF pathway in transmitting signals between various cell populations. d) SEM image showing a TNT (white arrows) between macrophages (green) and BMSC (blue). e) SEM image showing the interaction between the TNT and macrophages (red arrows). Yellow arrows show the buds from the TNT that are fused with the macrophages. f) Representative confocal images showing TNT‐mediated transfer of Tom20‐GFP‐tagged mitochondria from macrophages to BMSC. Colocalization of extracellular proteins Sec3 (pink) and Sec5 (grey) within TNT. Actin was stained with phalloidin red. g) Immunofluorescence image showing the colocalization of Miro1 (pink) with Tom20‐GFP‐labeled mitochondria (green). Macrophages transfected with Tom20‐GFP were cocultured with BMSC. The coculture was fixed for 24 h and immunolabeled with an anti‐Miro1 antibody (pink). Phalloidin red was used to stain actin (red). The image shows the colocalization of mitochondria (green) and Miro1 (pink) in the TNT. h,i) Graph showing the reduced mitochondrial transfer after shRNA‐mediated partial knockdown of Miro1 in macrophages (h). Before incubation, macrophages were marked with Mitotracker Deep Red (red). Data are normalized to mitochondrial transfer in controls (i). *P < 0.05, **P < 0.01, ***P < 0.001.

Given the complex role of the immune microenvironment in tissue regeneration, it is crucial to gain a deeper understanding of the potential mechanisms regulating bone defect repair. Therefore, we conducted bioinformatics analysis using the single‐cell RNA sequencing (scRNA seq) dataset of in situ cells during bone repair (Figure S1a, Supporting Information).^[^ ^18^ ^]^ Regarding CellChat analysis, we found a close signal interaction between the cells of the Col1a labeled osteoblast Cell lineage and immune cells (Figure S1b–g, Supporting Information), of which the interaction with macrophages was the closest (Figure S1f–i, Supporting Information). Through the analysis of the macrophage‐mediated signal pathway, we found that secreted phosphoprotein 1 (SPP1), transforming growth factor β (TGF‐β), and growth differentiation factor (GDF) have effective targeting in osteoblastic lineage cells (**Figure** **2**a). Among them, macrophages were the primary signal senders, while osteoblast lineage cells are pivotal signal receivers in bone repair (Figure 2b,c and Figure S1j,k, Supporting Information). Macrophages are considered prominent participants in bone regeneration and the acute inflammatory response to biomaterials, mainly due to their high plasticity in response to environmental cues and their multiple roles in bone homeostasis.^[^ ^19^ ^]^

To explore new mechanisms of regulation between macrophages and BMSC, we established a simple experiment using scanning electron microscopy (SEM) to examine the intercellular interactions between macrophages and BMSC in co‐culture. The SEM images exhibited that macrophages and BMSC were connected through tunneling nanotubes (TNT) (Figure 2d), as well as multiple contacts were formed on the membrane of macrophages (Figure 2e). We performed immunolabeling on the co‐culture of macrophages and BMSC, revealing the co‐localization of extracellular complexes Sec3 and Sec5 in the recruitment site of the Actin Cytoskeleton in TNT (Figure 2f). Especially, the co‐localization of Tom20‐GFP‐labeled mitochondria in TNT was observed by using phalloidin red staining for F‐actin. Moreover, TNT has been proven to be an essential form of mitochondrial transmission between macrophages and BMSC, which was confirmed by relevant experiments such as transmission electron microscope (TEM), immunogold labeling, and flow cytometry (Figure S2a–f, Supporting Information).

In addition, immune labeling revealed the co‐localization of Tom20‐GFP‐labeled mitochondria and Miro1 in TNT (Figure 2g). A significant reduction in mitochondrial transfer was observed through partial knockout of Miro1 based on shRNA (Figure 2h,i), confirming Miro1‐mediated mitochondrial transfer from macrophages to BMSC. It has been pointed out that TNT is formed through cytoskeleton rearrangement activated by Ras GTPase, and Miro1 is involved in the active transport of mitochondria through TNT.^[^ ^20^ ^]^ Emerging evidence indicates mitochondria have dynamic properties and can achieve intercellular movement across cell boundaries.^[^ ^21^ ^]^ Mitochondrial transfer between cells helps to integrate the mitochondria of donor cells into the endogenous mitochondrial network of receptor cells, thus causing changes in the function of receptor cells. Numerous researchers have realized that mitochondrial transfer profoundly impacts cell differentiation and tissue homeostasis.^[^ ^22^ ^]^ It suggests that intercellular mitochondrial transfer represents an underappreciated cellular communication mechanism and plays an essential regulatory role in bone defect repair.

### 2.2 Engineering Hierarchical Hydrogel for Bone Regeneration Strategy

The initiation of bone defect repair is complex, involving dynamic interactions between various inflammations and mechanical reactions.^[^ ^23^ ^]^ Especially, the significance of the immune microenvironment in the early repair stage, the complexity and correlation of the bone defect repair process, and the mechanical interactions of related cells.^[^ ^24^ ^]^ To better adapt to calvarial defects, the designed hierarchical hydrogel has numerous unique properties, including suitable mechanical properties, stimuli‐responsive performance, injectability, self‐healing ability, and biodegradability.

**Figure 3:**
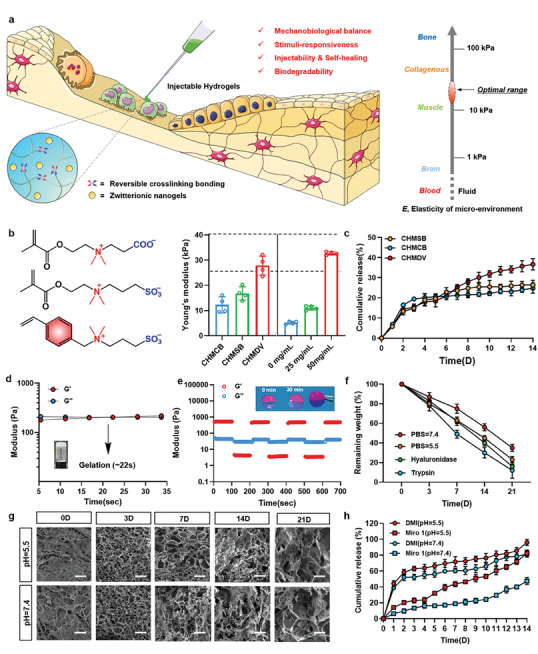
Engineering hierarchical hydrogel for bone regeneration strategy. a) Schematics elucidating the fundamental biological prerequisites for implanted injectable hydrogel featuring reversible crosslinking bonding for tissue engineering. The inset illustrates the preferable elastic modulus (*E*, measured in kPa) of injectable hydrogel juxtaposed with that of animal tissues and organs. b) Young's modulus of different hierarchical hydrogels (CHMCB, CHMSB, and CHMDV), as well as CHMDV with different contents of MDV nanogels (0, 25, and 50 mg mL^−1^). The inset shows the chemical structures of CBMA, SBMA, and DVBAPS (*n* = 3). c) Release profiles of rhodamine B from the hierarchical hydrogels at 37 °C in normal saline (0.9 wt%). Rheological analysis of d) short timescale gelation behavior and e) continuous step strain sweeping of hierarchical hydrogel (*n* = 3). f) Degradation rate‐time curves of hierarchical hydrogels in trypsin, hyaluronidase, and phosphate‐buffered saline (PBS, pH = 5.5 and 7.4). (*n* = 3). g) Time‐dependent SEM images of dried hierarchical hydrogels and h) the release profiles of the DMI and Miro 1 under different pH environments (*n* = 3). Scale bar, 20 µm (Day 0) and 100 µm (Day 3–15).

The hierarchical hydrogel consisted of two parts, one part is an injected hydrogel framework constructed from chitosan and aldehyde‐modified sodium hyaluronate (OSH), and the other part is macrophage‐targeted zwitterionic nanogel (**Figures** **3**a and S3, Supporting Information). Encouraged by the promising role of zwitterionic nanogels species and content in determining the mechanical strength, we constructed hierarchical hydrogel with suitable mechanical properties to offer an appropriate extracellular matrix (ECM) environment for in situ bone healing and regeneration (Young's modulus ranging from 25–40 kPa).^[^ ^25^ ^]^ First, due to the structural variety of zwitterions, three representative monomers (i.e., CBMA, SBMA, and DVBAPS) were employed for the dispersion polymerization, and we synthesized three different structurally mannose‐modified zwitterionic nanogels (i.e., MCB, MSB, or MDV nanogels) (Figure S4, Supporting Information). Subsequently, three representative zwitterionic nanogels were introduced into the hydrogel framework, to enhance the mechanical strength of the hydrogel. These three hierarchical hydrogels were named, respectively, CHMCB, CHMSB, and CHMDV. Young's modulus of CHMDV was validated by a compressive test (27.81 ± 3.23 kPa), which was approximately 2.3 times and 1.6 times stronger than CHMCB (12.26 ± 2.77 kPa) and CHMSB (16.78 ± 2.30 kPa). We further speculate that the rigid structure of the benzene group could significantly enhance the mechanical properties of nanogel compared to the ester group, further changing the mechanical properties of hierarchical hydrogels. Besides, hierarchical hydrogel with varied MDV nanogel loading confirmed that the proper mechanical strength of the hydrogel needs to be introduced at specific concentrations of MDV nanogels (≥50 mg mL^−1^). Therefore, hierarchical hydrogel with suitable MDV nanogels loading has been demonstrated to stimulate the expression of osteogenic markers (Figure 3b and Figure S5, Supporting Information).

The dye‐releasing profiles of hierarchical hydrogels were also investigated to demonstrate the stimulate‐responsive property of nanogels (Figure 3c). Results revealed that MDV nanogels exhibited a higher percentage of release (36.7%) as compared to MCB nanogels (24.8%) and MSB nanogels (26.4%) in normal saline (0.9% wt) at 37 °C. Therefore, we further investigated the properties of the hydrogel with MDV nanogels. The injection capacity of the hierarchical hydrogel is thoroughly assessed through an oscillatory time sweep, and the sol‐gel transition time is found to be ≈22 s, maintaining its shape within a brief period (Figure 3d). Moreover, the self‐healing rheological test demonstrates that the hierarchical hydrogel could fully recover its original value as the strain varies between 1% and 300% multiple times, derived from reversible Schiff base bonding (Figure 3e). The photographs and the lap‐shear tests showed that the hierarchical hydrogel had good adhesion to a variety of substrates (Figure S6a, Supporting Information). In vitro, degradation experiments illustrated that the degradation efficiency followed the order of trypsin, hyaluronidase, and phosphate‐buffered saline (PBS, pH = 5.5 and 7.4), with corresponding degradation rates of ≈88%, ≈84%, ≈77%, and ≈65% on day 21 (Figure 3f and Figure S6b, Supporting Information). SEM images demonstrated a gradual transition of pore structure from compact to lose within the hydrogel over time, the degradation process was primarily initiated from the inner part of the hydrogel (Figure 3g). We continued to examine the pH‐responsive property and gradient release of hierarchical hydrogel (Figure 3h and Figure S6c–f, Supporting Information). The anti‐inflammatory drug dimethyl itaconate (DMI) was loaded into the hydrogel framework and Miro1 was loaded into MDV nonogels, respectively. This whole system was named Gel@MDI (Tabel 1). The release of both DMI and Miro1 at pH = 5.5 was higher than that at pH = 7.4. This suggests that polymer networks decomposed more severely in an inflammatory environment, promoting the release of DMI and nanogels load‐Miro1. This hierarchical hydrogel has a sophisticated incorporation of micro and macro design elements. The nanogels in the hydrogel are capable of targeting macrophages and releasing therapeutic substances, resulting in effective biological performance at the micro level.

### 2.3 MDV Nanogels Loaded with Miro1‐DNA Can Target Macrophages and Regulate Mitochondrial Transfer

**Figure 4:**
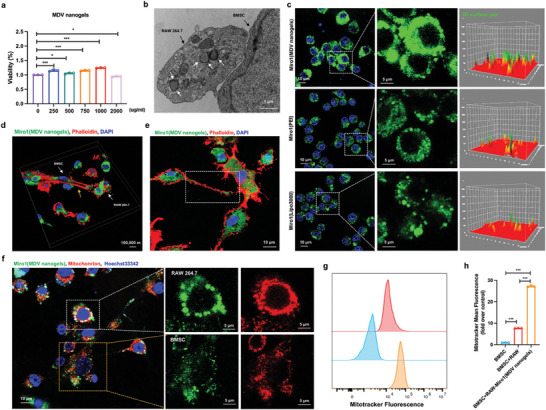
Mitochondrial transfer mediated by zwitterionic MDV nanogels. a) CCK‐8 analysis showing the concentration dependency on MDV nanogels (250–2000 µg mL^−1^) cocultured with macrophages for 24 h. b) SEM images showing the specific targeting of MDV nanogels towards macrophages (indicated by white arrows). c) Fluorescence images showing the transfection efficiency of the Miro1 plasmids by MDV nanogels, PEI, or Lipo3000. d,e) Immunofluorescence images showed that Miro1 and TNT were colocalized after the application of MDV nanogels. Actin was stained with phalloidin red. f) Representative confocal images showing Miro1 (MDV nanogels)‐mediated mitochondrial transfer from macrophages to BMSC. Before coculturing macrophages and BMSC, MitoTracker Deep Red was used to label macrophage mitochondria (red). g,h) The graph showing an increased mitochondrial transfer from macrophages to BMSC by the introduction of Miro1 (MDV nanogels). Monitored mitochondrial metastasis using flow cytometry and normalized data based on mitochondrial metastasis under control conditions. *P < 0.05, **P < 0.01, ***P < 0.001.

With the advantages of high biocompatibility, low cytotoxicity, minimal immunogenicity, high system stability, and long cycle time, zwitterion materials have become an ideal choice for designing therapeutic vectors. They are widely used as vectors for drug delivery and gene therapy.^[^ ^26^ ^]^ Therefore, we synthesized mannose‐modified zwitterionic nanogels (MDV nanogels) that have the characteristic of anti‐non‐specific protein adsorption and have been widely used in drug delivery systems and as tumor‐targeting carriers.^[^ ^27^ ^]^ MDV nanogels targeted at macrophages expressed low cytotoxicity at ≤2000 µg mL^−1^ on the macrophages (**Figure** **4**a), indicating good cell compatibility. To investigate the selective targeting ability of MDV nanogel on macrophages, we added MDV nanogel of 1000 µg mL^−1^ working concentration to the co‐culture system of macrophages and BMSC. Through TEM images, it could be found that MDV nanogels were present in macrophages rather than BMSC cells (Figure 4b). This suggests that MDV nanogels were selectively taken up by macrophages in this co‐culture system, indicating excellent macrophage targeting activity.

We further explored the prospects of zwitterion materials as gene therapy vectors. Mainly when utilized as a gene transfection vector, zwitterion displays reduced cytotoxicity compared to traditional cationic gene vectors while maintaining its capacity to transport gene molecules and higher cellular uptake efficiency. This presents a promising avenue for gene therapy research and applications.^[^ ^28^ ^]^ The Miro1‐DNA was introduced into MDV nanogels to bind and immobilize it with N^+^ cation activation. Simultaneously, the zwitterion demonstrated outstanding anti‐nonspecific adhesion capabilities, effectively protecting the Miro1‐DNATransfection of Miro1‐DNA was performed using traditional transfection reagents PEI, Lipo3000, and MDV nanogels, respectively. The expression of green fluorescent GFP on Miro1‐DNA was confirmed through ImageJ analysis of fluorescence intensity, and the results indicated that MDV nanogels exhibited higher transfection efficiency of Miro1‐DNA compared to traditional transfection methods (Figure 4c).

Mitochondrial transfer, as a repair strategy, has been proven to promote the survival and recovery of damaged cells.^[^ ^7a^ ^]^ Intercellular mitochondrial transfer not only saves tissue damage in the cardiovascular system, central nervous system, and respiratory system but also affects the function of tumor treatment resistance and inflammation regulation.^[^ ^29^ ^]^ However, under most pathological conditions, the efficiency of this self‐repair pathway usually needs to be improved.^[^ ^30^ ^]^ We investigated the relationship between Miro1 (MDV nanogels) and the mitochondrial transfer efficiency of macrophages. Introducing Miro1 (MDV nanogels) to the coculture system for 24 h, we observed a large amount of green fluorescence (Miro1) in macrophages (Figure 4d,e). Subsequently, we labeled the mitochondria of macrophages with MitoTracker Deep Red, washed them to remove any unbound dyes, and cocultured them with BMSC. After adding Miro1 (MDV nanogels) to the coculture for 24 h, a large number of mitochondria (red) from macrophages appeared in BMSC (Figure 4f). Moreover, we conducted flow cytometry analysis to measure the efficacy of mitochondrial transfer from macrophages to BMSC. Our findings revealed that Miro1 (MDV nanogels) demonstrated a statistically significant increase in mitochondrial transfer efficiency compared to the control group (Figure 4g,h). We confirmed through SEM and immunofluorescence that Miro1 mediates mitochondrial transfer between macrophages and BMSC through TNT. Utilizing MDV nanogels as gene carriers to achieve Miro1‐DNA targeted entry into macrophages improves mitochondrial transfer efficiency.

### 2.4 Gel@MDI Regulates the Osteogenic Differentiation Ability of BMSC by Regulating Their Energy Metabolism

Next, we evaluated the influence of hierarchical hydrogel on the polarity of macrophages and the efficiency of mitochondrial transfer. First, Gel@MDI did not affect the cellular activity of bone marrow stromal cells and has good biocompatibility via cytotoxicity assays (Figure S7a, Supporting Information). The hierarchical hydrogel constructed by the reversible dynamic covalent bond (─N═CH─) could effectively induce the release of the anti‐inflammatory drug DMI in an inflammatory environment. As a metabolite, DMI has been confirmed to play a regulatory role in inflammatory reactions. We confirmed that DMI released from Gel@MDI could significantly reduce the proportion of macrophages to M1‐like polarization induced by Lipopolysaccharide (LPS) (Figure S7b, Supporting Information). A significant increase in ROS in M1‐like macrophages was observed using 2‘,7′–dichlorodihydrofluorescein diacetate (DCFH‐DA). Excessive ROS increases mitochondrial permeability, leading to the release of cytochrome C and irreversible damage to mitochondria.^[^ ^31^ ^]^ Compared to the Gel group, the proportion of ROS in macrophages was significantly reduced in the Gel@MDI group (Figure S7c,d, Supporting Information). The release and action of DMI in Gel@MDI can effectively reverse the polarization of macrophages towards M1‐like and reduce the level of ROS in macrophages. The strong hydrophilicity of MDV nanogels can also reduce ROS and cell apoptosis to a certain extent.^[^ ^32^ ^]^

**Figure 5:**
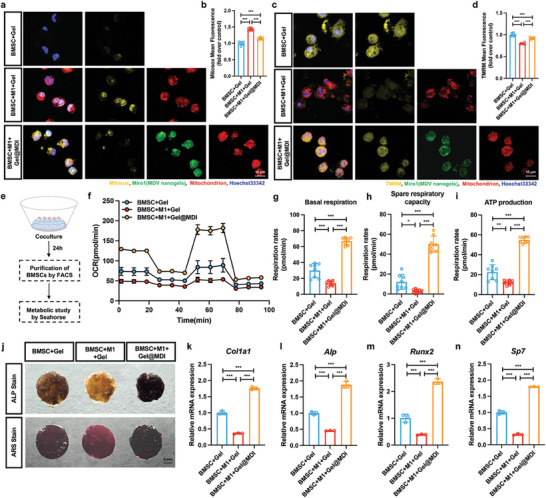
Metabolic effect of mitochondrial transfer. a) Fluorescence image showing the ROS level of BMSC on the hierarchical Gel@MDI hydrogel. MitoSox staining (yellow) indicates the ROS levels in BMSC. MitoTracker Deep Red labeled macrophage mitochondria (red) before coculture. b) The mean value of the intensity of the fluorescent markers for the quantification of ROS production (*n* = 6). c,d) Representative confocal images and corresponding analysis to quantify the MMP levels in BMSC on Gel@MDI. TMRM staining reflects MMP levels (*n* = 6). e) Schematic showing the metabolic analysis of the hippocampal platform to investigate the impact of mitochondrial transfer on BMSC. After coculture on the Gel@MDI hydrogel for 24 h, macrophages and BMSC were isolated by FACS, and the seahorse xfe96 platform was further employed to measure the metabolic status of BMSC. f) The oxygen consumption rate (OCR) of BMSC from the Seahorse XF Mito stress test showed increased mitochondrial respiration (*n* = 8). g) Basal respiration, h) spare respiratory capacity, and i) ATP production in the BMSC (*n* = 8). j) ALP staining and Alizarin Red S staining of BMSC cultured on the CH (pure Gel) or hierarchical hydrogels (Gel@MDI) 7 and 14 d after osteogenic differentiation. k–m) Expression of osteogenic related genes *Col1a1*, *Alp, Runx2*, and *Sp7* 7 d after osteogenic differentiation (*n* = 3). *P < 0.05, **P < 0.01, ***P < 0.001.

We then measured ROS and mitochondrial membrane potential (MMP) levels in BMSC to explore the effect of M1‐like macrophages on the metabolic status of BMSC under Gel@MDI intervention. According to MitoSOX, the ROS in mitochondria was measured, and M1‐like macrophages in ordinary hydrogel increased the ROS level in BMSC by transferring the oxidative damaged mitochondria (**Figure** **5**a,b). In addition, it was confirmed through tetramethylrhodamine methyl ester (TMRM) staining that M1‐like macrophages could cause a decrease in MMP levels in BMSC (Figure 5c,d), indicating mitochondrial dysfunction in BMSC. Under the action of Gel@MDI, we found that the mitochondrial function of BMSC was partially restored, manifested by a decrease in ROS levels and an increase in MMP levels (Figure 5a–d). We further utilized the metabolic analysis of the hippocampal platform to investigate the impact of the mitochondrial transfer on BMSC (Figure 5e). BMSC isolated from Gel@MDI showed higher basal respiration, spare respiratory capacity, and adenosine triphosphate (ATP) production than the CH hydrogel (Gel) group (Figure 5e–i).

The mitochondrial function of BMSC is crucial for their proliferation, migration, and differentiation.^[^ ^33^ ^]^ After the differentiation process begins, the mitochondria in BMSC are activated, and oxidative phosphorylation becomes the primary source of ATP.^[^ ^11b^ ^]^ This bioenergy switch is particularly important for the osteogenic differentiation of BMSC.^[^ ^34^ ^]^ The ATP level in osteoblasts increased with the morphogenesis of mineralized nodules during osteoblastic differentiation.^[^ ^35^ ^]^ In addition, Pietila et al. showed that mitochondrial membrane potential decreased during osteogenic differentiation.^[^ ^36^ ^]^ The levels of proteins involved in mitochondrial biogenesis, such as PGC‐1α, mtTFA, TCA cycle enzymes, and protein subunits of respiratory enzymes, also increase significantly after osteogenesis induction of BMSC.^[^ ^37^ ^]^ The up‐regulated mitochondrial function can provide greater energy demand to promote other biochemical reactions in cells.^[^ ^38^ ^]^ These results indicated that Gel@MDI could enhance the metabolic status of BMSC by influencing the polarity of macrophages and mitochondrial transmission. In order to more directly determine that the enhanced metabolism of BMSC can promote their osteogenic potential, we replaced the culture medium for osteogenic induction after cocultivation for 24 h. CH hydrogel is prepared with chitosan and aldehyde‐modified sodium hyaluronate as a Gel group. Compared to the BMSC+Gels group, the BMSC+M1+Gels group has a lower osteogenesis phenotype, which is manifested by lower alkaline phosphatase (ALP) levels, fewer mineralized nodules, and reduced osteogenesis‐related gene expression after 7 and 14 d of osteogenic induction (Figure 5j–n). In contrast, the BMSC+M1+ Gel@MDI group was able to significantly restore the osteogenic ability of BMSC, such as increased ALP levels, increased mineralization nodules, and increased expression of collagen type I alpha 1 chain (*Col1a1*), *Alp*, runt‐related transcription factor 2 (*Runx2*), and Sp7 transcription factor 7 (*Osx*) after 7 days of osteogenic induction.

### 2.5 Hierarchical Hydrogel with Immune Regulation and Targeted Mitochondrial Transfer Promoted the Regeneration of Critical‐Size Calvarial Defects

**Figure 6:**
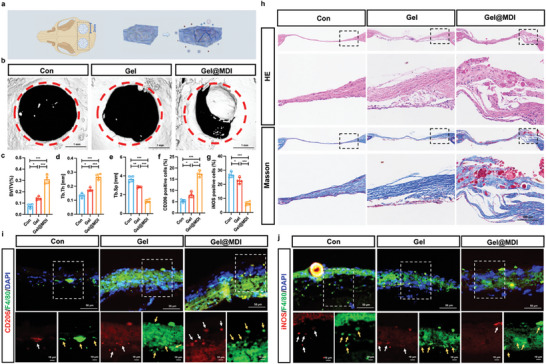
Transplantation of Gel@MDI promotes regeneration of critical‐sized bone defect. a) Illustration of the implantation of hydrogel after calvarial defect surgery. b–e) Micro‐CT evaluation of bone regeneration in surgery only (Con), CH (pure Gel), and hierarchical hydrogel (Gel@MDI) groups at 2 weeks postsurgery, respectively. Quantitative analysis of newly regenerated bone tissues, including c) BV/TV, d) Tb.Th, and e) Tb.Sp (*n* = 3). Quantitative analysis of f) CD206 positive staining areas and g) iNOS positive staining areas (*n* = 4). h) H&E staining and Masson' trichromic staining of paraffin sections in Con, Gel, or Gel@MDI groups at 8 weeks after calvarial defect. i,j) Coimmunofluorescent staining images of iNOS positive staining areas and CD206 positive staining areas (*n* = 4). *P < 0.05, **P < 0.01, ***P < 0.001.

We utilized mitochondrial transfer between macrophages and BMSC to achieve deep regulation of the immune microenvironment during bone defect repair by regulating energy metabolism and function of BMSC. To further evaluate the osteogenic properties of hydrogel in vivo and better repeat the results in vitro, we established a critical‐size calvarial defect model in mice. Eight weeks after the hydrogel implantation, micro‐computed tomography (micro‐CT) scanning images (**Figure** **6**a,b) were performed. Gel@MDI group consisted of two parts, one part was an injected hydrogel framework constructed from chitosan and aldehyde‐modified sodium hyaluronate (OSH), and the other part was macrophage‐targeted zwitterionic nanogels (MDV nanogels). Moreover, the anti‐inflammatory drug dimethyl itaconate (DMI) was loaded into the hydrogel framework and Miro1 was loaded into MDV nonogels. Compared to the Gel group, the Gel@MDI group showed significantly enhanced bone regeneration, characterized by more calcification and complex tissue formation in the calvarial defect area (Figure 6a and Figure S8a, Supporting Information). In the Gel@MDI group, bone tissue volume/total tissue volume (BV/TV) and trabecular thickness (Tb. Th) were significantly higher than in other groups, and trabecular separation/spacing (Tb. Sp) was significantly lower (Figure 6c–e). When the key components DMI, MDV nanogels, and Miro1‐DNA in hydrogel are present/absent, bone formation is less than that in the Gel@MDI group (Figure S8a, Supporting Information). The same results were obtained through Micro‐CT analysis, which confirmed that the addition of DMI to change the polarity of macrophages, the introduction of zwitterionic nanogels to change the hardness of hydrogel, or the addition of Miro1‐DNA to promote mitochondrial transfer could promote the repair of calvarial defects to some extent (Figure S8b–d, Supporting Information).

By analyzing and comparing the new bone in the Gel@I group and Gel@MDI group, it can be proved that this graded hydrogel mainly affects bone regeneration by regulating the transfer of mitochondria between cells (Figure S8, Supporting Information). The regulation of macrophage polarity by DMI can have an anti‐inflammatory effect, possibly due to the activation of some paracrine signaling pathways, which cannot completely exceed the effect of Gel@MDI on bone regeneration and repair. The addition of MDV nanoparticles is not only to increase the expression of Miro1 in macrophages but also to promote mitochondrial transfer efficiency. It can provide certain mechanical properties and provide suitable conditions for bone repair and regeneration.

In addition to analyzing the increase in bone mass, we also evaluated the histological structure and immune status of the new bone tissue. Hematoxylin eosin staining (H&E) and Masson's trichromic staining showed that the newly formed bones were evenly distributed and staggered in the Gel@MDI group compared to the other two groups (Figure 6h). We determined the polarity of macrophages in repaired bone tissue by immunofluorescence of macrophages with the M1/M2‐like markers iNOS and CD206. The experiment found that Gel@MDI promoted the expression of the M2‐like macrophage marker CD206 (Figure 6f–i) and reduced the expression of the M1‐like macrophage marker iNOS in bone repair tissues (Figure 6g,j). The experimental results indicated that the patterns of the initial DMI release and subsequent sustained Miro1‐DNA release in the Gel@MDI could regulate immune and mitochondrial metastasis, promoting calvarial tissue regeneration.

**Figure 7:**
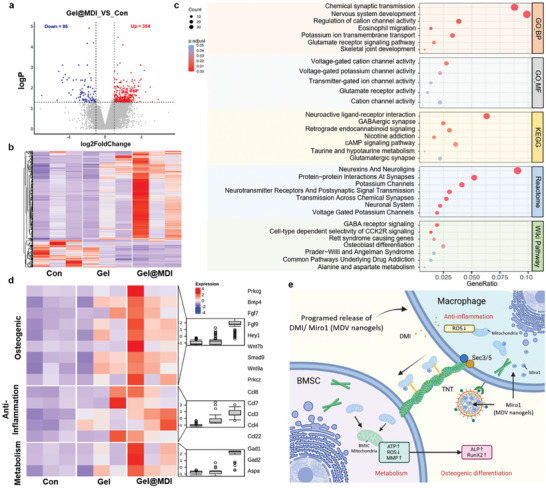
Exploration of biological processes related to mitochondrial transfer regulation in tissue regeneration. a) Volcano plot showing differentially expressed genes between the surgery only (Con) and hierarchical hydrogel (Gel@MDI) groups. b) Heat map of gene expression among surgery only (Con), CH hydrogel (Gel), and hierarchical hydrogel (Gel@MDI) groups. c) Biological process (BP), molecular function (MF), KEGG, reactome, and Wiki pathway enrichment analysis revealed potential pathways in which differentially expressed genes may participate. d) Heat map and box plot analysis of “Osteogenic,” “Anti‐inflammation,” and “Metabolism” pathways. e) Schematic showing the possible mechanism of promoting osteogenesis in the Gel@MDI‐induced BMSC.

In vivo experiments demonstrated that the hierarchical Gel@MDI showed strong bone induction and repair capabilities. To delve deeper into the underlying mechanism of Gel@MDI‐induced tissue regeneration and associated biological processes, we collected tissues from mouse calvarial bone defects that were either left untreated (control), treated with CH hydrogel (Gel), or treated with hierarchical hydrogel (Gel@MDI) 8 weeks postsurgery, for subsequent biological information analysis (Figure S9a, Supporting Information). We utilized DeSeq2 to conduct differential expression analysis, revealing that the most substantial difference exists between the Gel@MDI and control groups, as illustrated in Figure S9b (Supporting Information). Consequently, through a comparison between the Gel@MDI and control groups, we pinpointed 449 differentially expressed genes (*p* < 0.05&| log2 fold change |>1), which were visually represented using heat maps, comprising 354 upregulated genes and 95 downregulated genes (**Figure** **7**a,b). We also performed enrichment analysis for these differentially expressed genes in GO, KEGG, Reactome, and Wiki Pathway databases. These differentially expressed genes were enriched in pathways related to nerve regeneration, osteogenic differentiation, and metabolism (Figure 7c). Consistently, gene set enrichment analysis (GSEA) revealed significant gene enrichment in “Bone maturation” and “Bone removal,” further confirming the role of Gel@MDI in bone defect repair (Figure S9c–g, Supporting Information). Subsequently, we performed heatmaps and box plots on critical pathways. Compared to the control group and the Gel group, the expression of osteogenic, anti‐inflammatory, and metabolic genes increased in the Gel@MDI group (Figure 7d). These results suggest that the Gel@MDI group played a role in developing the nervous system, migration of eosinophils, bone development and other inflammatory reactions, and bone regeneration. The above results proved that our hierarchical hydrogel has extraordinary immune response, metabolic regulation, and bone induction properties, which benefit bone immune regulation (Figure 7e).

## 3 Conclusion

This study developed a hierarchical hydrogel with dual immune and bioenergy metabolism regulation, demonstrating the therapeutic potential of mitochondrial replenishment therapy in tissue engineering such as bone regeneration. The engineered hierarchical hydrogel (Gel@MDI) provides a mechanical sensing environment conducive to osteogenesis and regulates mitochondrial metabolism through intercellular mitochondrial transfer. In the initial stage of bone defect repair, this hydrogel shows an inflammatory response and promotes immune regulation. Subsequently, the targeting and high transfection ability of MDV nanogel increased the mitochondrial transfer rate between macrophages and BMSC, thus affecting the mitochondrial metabolism of BMSC. Our study emphasizes the importance of mutual environmental regulation in bone defect repair, opening up new avenues for tissue regeneration engineering.

## 4 Experimental Section

### Materials

Minimum essential medium α (α‐MEM), Dulbecco's modified Eagle's medium (DMEM), fetal bovine serum (FBS), penicillin‐streptomycin, MitoTracker Deep Red, and RNAeasy Mini Kit were purchased from Invitrogen Co., Ltd. (Carlsbad, USA). Sec3, Sec5, F4/80, iNOS, CD206 antibody, and the secondary antibody Proteintech Co., Ltd. (Chicago, USA). Dimethyl itaconate (DMI), 3‐[dimethyl‐[2‐(2‐methylprop‐2‐enoyloxy)ethyl]azaniumyl]propane‐1‐sulfonate (SBMA), and 3‐[[2‐(methacryloyloxy)ethyl]dimethylammonio]propionate (CBMA) were obtained from Aladdin Reagent Co., Ltd. (Shanghai, China). Dimethyl‐(4‐vinylphenyl)ammonium propane sulfonate (DVBAPS) and aldehyde‐modified sodium hyaluronate (OSH) were synthesized following previously reported methods.^[^ ^39^ ^]^ Other uncommercialized monomers were synthesized and characterized in the Supporting Information.

### Synthesis of Zwitterionic Nanogels

Zwitterionic nanogels were typically synthesized using dispersion polymerization, following the procedure outlined below.^[^ ^40^ ^]^ In a typical synthesis, DVBAPS (2.0 g), AH (0.2 g), PVP (0.4 g), AIBN (15 mg), and 21 g of water/ethanol (30/70, w/w) were added in the 100 mL flask and thoroughly mixed. After vigorously stirring the solution and deoxygenating it by bubbling nitrogen gas for 30 min, the mixture was subsequently transferred to a water bath at 50 °C. Following the nucleation step, the solution of cross‐linker BAC (28 mg) in 9 g water/ethanol (30/70 w/w) medium was added dropwise for 2 h by a syringe pump. The reaction was allowed to proceed overnight. Subsequently, the dispersion was centrifuged and subjected to multiple washes with ethanol to thoroughly remove any residual monomers and stabilizers. The other zwitterionic nanogels, namely CB and SB nanogels, were prepared using a similar method.

### Preparation of the Engineered Hierarchical Hydrogel Gel@DMI

In a typical synthesis, 100 mg CS was fully dissolved into a 1 mL PBS solution and denoted as solution I. Solution II was formulated by mixing OSH (60 mg mL^−1^), MDV nanogels loaded with Miro 1 (50 mg mL^−1^), and DMI (250 × 10^−6^ m) in a PBS solution. Afterward, solutions I and II were introduced into two separate barrels of a double‐barrel syringe. The two solutions were homogenized and then delivered to the targeted defect site using a specially designed mixing injection tip, obtaining the engineered hierarchical hydrogel Gel@DMI. The CH hydrogel (Gel) was prepared by CS and OSH via a similar process.

### Evaluation of Mitochondrial Labeling and Transfer Efficiency

Mitochondria of macrophages were transfected and labeled with Tom20‐GFP lentivirus or stained with 100 × 10^−6^ m MitoTracker Deep Red. Wash MitoTracker Deep Red stained cells three times and then incubate them in a complete culture medium for 1 h to ensure the removal of excess unbound MitoTracker dye. CytoFLEX LX flow cytometry (Beckmancoulter) further detected the efficiency of mitochondrial transfer between macrophages and BMSC. Coculture RAW 264.7 cell and BMSC or incubate in Transwell (0.4 µm pore polycarbonate membranes) for 24 h. Alternatively, investigate the effects of knocking out or overexpressing Miro1 on mitochondrial metastasis under shMrio1 and Miro1 (MDV nanogels) action. Using the macrophage marker F4/80‐PE‐Cy7 (1:400), it was determined that the MitoTracker marker in F4/80^−^ BMSC comes from macrophage mitochondria. Use FlowJo software to analyze the average fluorescence intensity of mitochondria received in BMSC.

### Metabolic Analysis by Seahorse

Metabolic analysis was performed using the XFe96 extracellular flux analyzer (Seahorse Bioscience). Processed BMSC (1.6 × 10^4^) inoculate cells onto an XFe96 well Seahorse microplate in complete culture medium. The medium in each well was replaced with XF assay buffer supplemented with 10 × 10^−3^ m glucose, 1 × 10^−3^ m sodium pyruvate, and 2 × 10^−3^ m L‐glutamine. Afterward, balance the plate at 37 °C and place it in a carbon dioxide‐free incubator for 1 h. Through the gradual injection of oligomycin (1 × 10^−6^ m), FCCP (1.0 × 10^−6^ m), and a mixture of rotenone (0.5 × 10^−6^ m) and antimycin A (0.5 × 10^−6^ m) conduct testing. Normalize the data based on the total protein concentration in each well. Data analysis was conducted using Seahorse Wave software 2.6.1.

### Calvarial Bone Defect Model

The research agreement was approved by the Ethics Committee of Zhejiang University ((No. ZJU20230294). The Experimental Animal Center of Zhejiang University provided the C57BL/6 mice (9 weeks, male) used in this study. All experimental procedures have been approved by Zhejiang University and conducted in accordance with laboratory animal care and usage guidelines. First, the mice were anesthetized with 1% pentobarbital sodium, and a linear incision was made in the middle area of the calvarial cap along the front and back directions. Two bone defects with a diameter of 3 mm were caused by circular drills located in different parietal regions. The mice were divided into three groups: i) defect control group (Con), ii) CH hydrogel group (Gel), and iii) hierarchical hydrogel group (Gel@MDI). The mice were euthanized 8 weeks after implantation, and their calvarias were harvested at room temperature and fixed with 4% paraformaldehyde for 24 h. Then, a micro‐computed tomography scan was performed to evaluate the new bone formation.

### Statistical Analysis

GraphPad Prism 9.0 (GraphPad Software, USA) was used for statistical analysis. All data were expressed as mean ± standard deviation, showing a continuous normal distribution. A one‐way analysis of variance (ANOVA) was used to determine differences among multiple groups. Two sample groups were analyzed using a two‐tailed Student's t‐test. The comparison between the two‐factor groups was first conducted through a two‐way ANOVA. A *p*‐value < 0.05 was considered statistically significant. *P < 0.05, **P < 0.01, ***P < 0.001.

## Conflict of Interest

The authors declare no conflict of interest.

## Supporting information

Supporting Information

## Data Availability

The data that support the findings of this study are available in the Supporting Information of this article.

## References

[1] A. Ceriello , L. Monnier , D. Owens , Lancet Diabetes Endocrinol. 2019, 7, 221. 30115599 10.1016/S2213-8587(18)30136-0

[2] a) X. Ding , J. Shi , J. Wei , Y. Li , X. Wu , Y. Zhang , X. Jiang , X. Zhang , H. Lai , Sci. Adv. 2021, 7, eabj7857; 34890238 10.1126/sciadv.abj7857 PMC8664252

[3] Z. Wang , Y. Wang , J. Yan , K. Zhang , F. Lin , L. Xiang , L. Deng , Z. Guan , W. Cui , H. Zhang , Adv. Drug Delivery Rev. 2021, 174, 504. 10.1016/j.addr.2021.05.007 33991588

[4] A. Salhotra , H. N. Shah , B. Levi , M. T. Longaker , Nat. Rev. Mol. Cell Biol. 2020, 21, 696. 32901139 10.1038/s41580-020-00279-w PMC7699981

[5] N. Su , C. Villicana , F. Yang , Biomaterials 2022, 286, 121604. 35667249 10.1016/j.biomaterials.2022.121604 PMC9881498

[6] S. K. Biswas , A. Mantovani , Nat. Immunol. 2010, 11, 889. 20856220 10.1038/ni.1937

[7] a) W. Cai , J. Zhang , Y. Yu , Y. Ni , Y. Wei , Y. Cheng , L. Han , L. Xiao , X. Ma , H. Wei , Y. Ji , Y. Zhang , Adv. Sci. 2023, 10, e2204871; 10.1002/advs.202204871 PMC9896036 36507570

[8] K. Ning , S. Liu , B. Yang , R. Wang , G. Man , D. E. Wang , H. Xu , Mol. Metab. 2022, 58, 101450. 35121170 10.1016/j.molmet.2022.101450 PMC8888956

[9] C. Zheng , J. Chen , S. Liu , Y. Jin , Int. J. Oral Sci. 2019, 11, 23. 31423011 10.1038/s41368-019-0060-3 PMC6802669

[10] J. C. Drake , R. J. Wilson , R. C. Laker , Y. Guan , H. R. Spaulding , A. S. Nichenko , W. Shen , H. Shang , M. V. Dorn , K. Huang , M. Zhang , A. B. Bandara , M. H. Brisendine , J. A. Kashatus , P. R. Sharma , A. Young , J. Gautam , R. Cao , H. Wallrabe , P. A. Chang , M. Wong , E. M. Desjardins , S. A. Hawley , G. J. Christ , D. F. Kashatus , C. L. Miller , M. J. Wolf , A. Periasamy , G. R. Steinberg , D. G. Hardie , et al., Proc. Natl. Acad. Sci. USA 2021, 118, e2025932118.34493662

[11] a) A. S. Monzel , J. A. Enríquez , M. Picard , Nat. Metab. 2023, 5, 546; 37100996 10.1038/s42255-023-00783-1 PMC10427836

[12] M. P. King , G. Attardi , Cell 1988, 52, 811. 3349520 10.1016/0092-8674(88)90423-0

[13] J. C. Chang , H. S. Chang , Y. C. Wu , W. L. Cheng , T. T. Lin , H. J. Chang , S. J. Kuo , S. T. Chen , C. S. Liu , J. Exp. Clin. Cancer Res. 2019, 38, 30. 30674338 10.1186/s13046-019-1028-z PMC6343292

[14] a) J. Sun , H. T. J. Lo , L. Fan , T. L. Yiu , A. Shakoor , G. Li , W. Y. W. Lee , D. Sun , Sci. Adv. 2022, 8, eabp9245; 35977014 10.1126/sciadv.abp9245 PMC9385153

[15] a) K. Hayakawa , E. Esposito , X. Wang , Y. Terasaki , Y. Liu , C. Xing , X. Ji , E. H. Lo , Nature 2016, 535, 551; 27466127 10.1038/nature18928 PMC4968589

[16] S. Sagar , M. I. Faizan , N. Chaudhary , V. Singh , P. Singh , A. Gheware , K. Sharma , I. Azmi , V. P. Singh , G. Kharya , U. Mabalirajan , A. Agrawal , T. Ahmad , S. Sinha Roy , Cell Death Dis. 2023, 14, 324. 37173333 10.1038/s41419-023-05810-3 PMC10181927

[17] S. Obuobi , A. Ngoc Phung , K. Julin , M. Johannessen , N. Škalko‐Basnet , Biomacromolecules 2022, 23, 303. 34914360 10.1021/acs.biomac.1c01274 PMC8753600

[18] X. Zhang , W. Jiang , C. Xie , X. Wu , Q. Ren , F. Wang , X. Shen , Y. Hong , H. Wu , Y. Liao , Y. Zhang , R. Liang , W. Sun , Y. Gu , T. Zhang , Y. Chen , W. Wei , S. Zhang , W. Zou , H. Ouyang , Nat. Commun. 2022, 13, 5211. 36064711 10.1038/s41467-022-32868-y PMC9445030

[19] a) J. Tuckermann , R. H. Adams , Nat. Rev. Rheumatol. 2021, 17, 608; 34480164 10.1038/s41584-021-00682-3 PMC7612477

[20] T. Saha , C. Dash , R. Jayabalan , S. Khiste , A. Kulkarni , K. Kurmi , J. Mondal , P. K. Majumder , A. Bardia , H. L. Jang , S. Sengupta , Nat. Nanotechnol. 2022, 17, 98. 34795441 10.1038/s41565-021-01000-4 PMC10071558

[21] S. Shanmughapriya , D. Langford , K. Natarajaseenivasan , Ageing Res. Rev. 2020, 62, 101128. 32712108 10.1016/j.arr.2020.101128 PMC7484258

[22] N. Tseng , S. C. Lambie , C. Q. Huynh , B. Sanford , M. Patel , P. S. Herson , D. R. Ormond , J. Cereb. Blood Flow Metab. 2021, 41, 761. 32501156 10.1177/0271678X20928147 PMC7983509

[23] S. Debnath , A. R. Yallowitz , J. McCormick , S. Lalani , T. Zhang , R. Xu , N. Li , Y. Liu , Y. S. Yang , M. Eiseman , J. H. Shim , M. Hameed , J. H. Healey , M. P. Bostrom , D. A. Landau , M. B. Greenblatt , Nature 2018, 562, 133. 30250253 10.1038/s41586-018-0554-8 PMC6193396

[24] a) J. Zhang , D. Tong , H. Song , R. Ruan , Y. Sun , Y. Lin , J. Wang , L. Hou , J. Dai , J. Ding , H. Yang , Adv. Mater. 2022, 34, 2202044; 10.1002/adma.202202044 35785450

[25] A. Sánchez‐Ferrero , Á. Mata , M. A. Mateos‐Timoneda , J. C. Rodríguez‐Cabello , M. Alonso , J. Planell , E. Engel , Biomaterials 2015, 68, 42. 26264645 10.1016/j.biomaterials.2015.07.062

[26] Y. Zhou , Z. Chen , D. Zhao , D. Li , C. He , X. Chen , Adv. Mater. 2021, 33, 2102044. 10.1002/adma.202102044 34216408

[27] a) L. Zhang , Z. Cao , T. Bai , L. Carr , J. R. Ella‐Menye , C. Irvin , B. D. Ratner , S. Jiang , Nat. Biotechnol. 2013, 31, 553; 23666011 10.1038/nbt.2580

[28] J. B. Schlenoff , Langmuir 2014, 30, 9625. 24754399 10.1021/la500057j PMC4140545

[29] D. Liu , Y. Gao , J. Liu , Y. Huang , J. Yin , Y. Feng , L. Shi , B. P. Meloni , C. Zhang , M. Zheng , J. Gao , Signal Transduction Targeted Ther. 2021, 6, 65. 10.1038/s41392-020-00440-z PMC7884415 33589598

[30] S. P. Burr , F. Klimm , A. Glynos , M. Prater , P. Sendon , P. Nash , C. A. Powell , M. L. Simard , N. A. Bonekamp , J. Charl , H. Diaz , L. V. Bozhilova , Y. Nie , H. Zhang , M. Frison , M. Falkenberg , N. Jones , M. Minczuk , J. B. Stewart , P. F. Chinnery , Cell 2023, 186, 1212. 36827974 10.1016/j.cell.2023.01.034

[31] A. G. Manford , E. L. Mena , K. Y. Shih , C. L. Gee , R. McMinimy , B. Martínez‐González , R. Sherriff , B. Lew , M. Zoltek , F. Rodríguez‐Pérez , M. Woldesenbet , J. Kuriyan , M. Rape , Cell 2021, 184, 5375. 34562363 10.1016/j.cell.2021.09.002 PMC8810291

[32] T. Bai , J. Li , A. Sinclair , S. Imren , F. Merriam , F. Sun , M. B. O'Kelly , C. Nourigat , P. Jain , J. J. Delrow , R. S. Basom , H. C. Hung , P. Zhang , B. Li , S. Heimfeld , S. Jiang , C. Delaney , Nat. Med. 2019, 25, 1566. 31591594 10.1038/s41591-019-0601-5

[33] C. X. Zheng , B. D. Sui , X. Y. Qiu , C. H. Hu , Y. Jin , Trends Mol. Med. 2020, 26, 89. 31126872 10.1016/j.molmed.2019.04.008

[34] B. Li , Y. Shi , M. Liu , F. Wu , X. Hu , F. Yu , C. Wang , L. Ye , Stem Cell Res. Ther. 2022, 13, 77. 35193674 10.1186/s13287-022-02748-9 PMC8864833

[35] W. C. Lee , A. R. Guntur , F. Long , C. J. Rosen , Endocr. Rev. 2017, 38, 255. 28472361 10.1210/er.2017-00064 PMC5460680

[36] M. Pietilä , S. Palomäki , S. Lehtonen , I. Ritamo , L. Valmu , J. Nystedt , S. Laitinen , H. V. Leskelä , R. Sormunen , J. Pesälä , K. Nordström , A. Vepsäläinen , P. Lehenkari , Stem Cells Dev. 2012, 21, 575. 21615273 10.1089/scd.2011.0023 PMC3280604

[37] X. Li , X. Wang , C. Zhang , J. Wang , S. Wang , L. Hu , Cell Proliferation 2022, 55, e13191. 35088483 10.1111/cpr.13191 PMC8891618

[38] Q. Li , Z. Gao , Y. Chen , M. X. Guan , Protein Cell 2017, 8, 439. 28271444 10.1007/s13238-017-0385-7 PMC5445026

[39] Y. Gu , Y. Yang , J. Yuan , Y. Ni , J. Zhou , M. Si , K. Xia , W. Yuan , C. Xu , S. Xu , Y. Xu , G. Du , D. Zhang , W. Sun , S. Y. Zheng , J. Yang , Biomacromolecules 2023, 24, 3345. 37380981 10.1021/acs.biomac.3c00379

[40] M. Vatankhah‐Varnoosfaderani , M. Ina , H. Adelnia , Q. Li , A. P. Zhushma , L. J. Hall , S. S. Sheiko , Macromolecules 2016, 49, 7204.

