# Supplementary material for: An Engineered Hierarchical Hydrogel with Immune Responsiveness and Targeted Mitochondrial Transfer to Augmented Bone Regeneration
Source: Adv Sci (Weinh). 2024 Sep 11;11(42):2406287. doi: 10.1002/advs.202406287 (PMC11558138; doi:10.1002/advs.202406287)
Supplement: Supplementary file 1 — Supporting Information [file ADVS-11-2406287-s001.docx]

**Supporting Information**

**An Engineered Hierarchical Hydrogel with Immune Responsiveness and Targeted Mitochondrial Transfer to Augmented Bone Regeneration**

*Wenjin Cai ^a#^, Shihua Mao ^a, b#^, Ying Wang ^a^, Bicong Gao ^a^, Jiaying Zhao ^a^, Yongzheng Li ^a^, Yani Chen ^a^, Dong Zhang ^c^*, Jintao Yang ^b^*********, Guoli Yang ^a^********

Dr. W. Cai, B. Gao, Prof. Y. Wang, G. Yang

^a^Stomatology Hospital, School of Stomatology, Zhejiang University School of Medicine Zhejiang Provincial Clinical Research Center for Oral Diseases

Key Laboratory of Oral Biomedical Research of Zhejiang Province

Cancer Center of Zhejiang University

Engineering Research Center of Oral Biomaterials and Devices of Zhejiang Province

Hangzhou 310000, P. R. China.

Email: [guo_li1214@zju.edu.cn](mailto:guo_li1214@zju.edu.cn)

Dr. H. Mao, Prof. J. Yang

^b^ Zhejiang Key Laboratory of Plastic Modification and Processing Technology

College of Materials Science & Engineering

Zhejiang University of Technology

Hangzhou 310014, P. R. China.

Email: [yangjt@zjut.edu.cn](mailto:yangjt@zjut.edu.cn)

Dr. D. Zhang

^c^ The Wallace H. Coulter Department of Biomedical Engineering

Georgia Institute of Technology and Emory University

Atlanta, GA 30318, United States.

Email: [dzhang470@gatech.edu](mailto:dzhang470@gatech.edu)

**Experimental Section**

*Materials;* β‐glycerophosphate, puromycin, Alizarin Red S, and 4′,6-diamidino-2-phenylindole (DAPI) were purchased from Sigma-Aldrich Chemical Co. (St. Louis, USA). Hoechst 33342, Tetramethylrhodamine methyl ester (TMRM), and Transwell, Lipofectamine 3000 were purchased from Invitrogen Co., Ltd. (Carlsbad, USA). Bovine serum albumin (BSA), LPS, and glutaraldehyde were purchased from Solarbio Technology Co., Ltd. (Beijing, China). F4/80-PE-Cy7 and CD80-PE-Cy7 antibodies were purchased from BioLegend Inc. (San Diego, USA). Ascorbic acid, dexamethasone and branched polyethylene [PEI; MW = 25,000] were purchased from Sigma-Aldrich Chemical Co. (St. Louis, USA). Phalloidin-iFluor 647 Reagent was purchased from Abcam Co., Ltd. (Cambridge, UK). GFP antibody, multiple fluorescence immunohistochemistry (TSA), SYBR Green qPCR Mix, and RT Master Mix were purchased from ABclonal Technology Co., Ltd. (Wuhan, China). BCIP/NBT Alkaline Phosphatase Color Development Kit, DCFH-DA, and CCK-8 reagent were purchased from Beyotime Biotechnology Co., Ltd. (Shanghai, China). MitoSOX red mitochondrial superoxide indicator was purchased from Yeasen BioTECH Co., Ltd. (Shanghai, China). LR white resin was purchased from Ted Pella Co., Ltd. (California, USA). XFe96 well Seahorse microplate, XF assay buffer, and glucose, sodium pyruvate were purchased from Agilent Technologies Co., Ltd. (California, USA). L-glutamine, oligomycin, carbonyl cyanide-p-triflouromethoxyphenylhydrazone (FCCP), rotenone, and antimycin A were purchased from Agilent Technologies Co., Ltd. (California, USA). Sodium periodate (NaIO_4_, 99.5%) and 2,2'-azobis(2-methylpropionitrile) (AIBN) were obtained from Aladdin Reagent Co., Ltd. (Shanghai, China). Ethylene glycol (98%) was purchased from Rhawn Chemical Technology Co., Ltd. (Jiangsu, China). Miro1 antibody was purchased from Santa Cruz Biotechnology Inc. (Texas, USA). Allylamine hydrochloride (AH), N, N'-bis(acryloyl)cystamine (BAC), mannose (98%), and poly(vinylpyrrolidone) (PVP, Mw=58000 g/mol) were purchased from Macklin Chemistry Co., Ltd. Sodium hyaluronate (SH, Mw=1300 kDa) was sourced from Li Young Biotechnology Co., Ltd. Chitosan (CS, Mw < 10000 Da) was acquired from Jinhu Carapace Products Co., Ltd. (Shandong, China). Water used in these experiments was obtained from a Millipore water purification system. All other reagents and solvents were commercially obtained at pure analytical grade and used as received without purification.

*Macrophage-targeted mannose-loaded zwitterionic nanogels:* Macrophage-targeted zwitterionic nanogels were synthesized by conjugating mannose onto the zwitterionic nanogels via the ring-opening reaction^[39]^. DV nanogels (100 mg) and mannose (36 mg, 20 mmol) were added to 5 mL deionized water and stirred for 12 h at 40 °C. After the reaction, the solution was subjected to purification using dialysis (MWCO=3500 Da) against DI water for 48 h, followed by freezing drying to obtain solid white MDV nanogels. Afterward, a mixture containing 10 mg of MDV nanogels and 1.0 mL of Miro 1 aqueous solution (1 μL/mL) was magnetically stirred for 12 h at 25°C. The resulting nanogels were then subjected to centrifugation and rinsed with deionized water twice to remove the free Miro1.

*Characterizations*

*Structural characterization:* ^1^H-NMR spectra were performed on an ANANCE III (400 MHz) in D_2_O using tetramethylsilane as the internal reference. Fourier-transform infrared (FT-IR) spectroscopy transmission data were recorded on a Nicolet 6700 spectroscopy with resolution at 4 cm^-1^ and scans at 32. The morphology of hydrogel was observed by a Tescan Vega 3 SBH scanning electron microscope (SEM). The diameter of nanogel was measured by dynamic light scattering (DLS, NanoBrook-Omni).

*Rheological and compressive properties*: We employed the rheometer (DHR-2, TA Instruments) with a diameter of 25 mm to evaluate the storage modulus (G') and loss modulus (G") of hydrogels. To investigate the dynamic properties of hydrogels during the injection process, we conducted frequency sweep tests at 1 Hz. We explored the critical strain region by conducting strain amplitude sweep tests, with the strain amplitude ranging from 1% to 600%. (cylinder sample size: D=25 mm, h=2 mm). The compressive properties were recorded on an Instron MOD EL5567 (cylinder sample size: D=20 mm, h=5 mm).

*Releasing property*: To study the pH-responsive release behavior, the samples were incubated in 10 mL of PBS buffer (pH = 7.4 and 5.5) for up to 21 days. At predetermined time intervals, 2 mL of the extract solution was replaced by 2 mL of fresh corresponding buffers for further quantitative analysis. According to the standard curve, the accumulative release amount of DMI and Miro 1 was calculated. The concentrations of DMI or Miro 1 in the withdrawn samples were then measured using a UV-vis spectrophotometer (PerkinElmer LAMBDA 750) at λ=230 nm and λ=490 nm, respectively.

*Self-healing property*: Macroscopic and microscopic observations assessed the self-healing capacity of the hydrogels. The dynamic self-healing property of hierarchical hydrogels was observed by macroscopic images and recorded on a DHR-2 rheometer that switched the strain from 1% to 300% via the continuous step strain experiment. The two hydrogels were labeled with two different dyes in preparation and then cut in half post-gel formation. One of the hydrogels was intentionally gapped, followed by rejoining the two halves without any external assistance. The assembled hydrogel structure was allowed to rest at room temperature for 30 minutes and imaged for analysis

*The in vitro degradability property:* The *in vitro* degradability property of hierarchical hydrogels was referred to in relevant literature^1^.The in vitro degradability of composite hydrogels was assessed by immersing them in different solutions, including PBS (pH=7.4 and 5.5), 0.25% trypsin solution, and 0.1% hyaluronidase solution. The dried composite hydrogels were placed in a 12-well plate, and each well was filled with 2 mL of diverse solution. Subsequently, the samples were all cultured in an incubator at 37 °C, with daily replacement of the culture medium. The samples were regularly collected, dried to a fully dehydrated state, and weighed while the degradation rate percentage of weight lost relative to the original hydrogel sample was calculated.

Degradation Rate (%)=$\frac{W_{0}-W_{d}}{W_{0}}\times100\%$

Where *W_0_* (g) is the initial dry weight of the composite hydrogel and *W_d_* (g) is the dry weight of the hydrogel measured after different incubation times.

*Cell culture:* BMSC were initially isolated from the tibias and femurs of mice. BMSC and RAW 264.7 cells were cultured in α-MEM and DMEM containing 10% FBS and 1% penicillin-streptomycin at 37 °C and 5% CO_2_. Evaluate cell proliferation using the CCK-8 assay. For the cytotoxicity of MDV nanogels, RAW 264.7 cells at a density of 10×10^4^ cells per well and 250-2000 µg/mL of MDV nanogels were inoculated into a 24-well plate. For the co-culture of cells and Gel@MDI, BMSCs were seeded at a density of 4 × 10^4^ cells mL^−1^ in 24‐well plates and cocultured with RAW 264.7 cells (10×10^4^ cells per well) for 24 h. Subsequently, the cells were incubated with CCK-8 reagent for 2 h, and the absorbance was measured at a wavelength of 450 nm. To evaluate the osteogenic differentiation of BMSC, cells were induced in the osteogenic medium containing 5 mM β‐glycerophosphate, 50 µg/mL ascorbic acid, and 100 nM dexamethasone.

*Lentivirus and plasmid construction:* Tom20-GFP lentivirus, sh-control lentivirus, and shMiro1 lentivirus were constructed by Generay Technologies (Shanghai, China). The HEK293T cells were transfected with branched polyethylene [PEI; MW=25000] and viral packaging constructs pMD2.G and pSPAX2 to produce lentivirus, and the virus supernatant was harvested 24 and 48 h after transfection. The lentivirus was transfected into RAW 264.7 for 72 h, and the selection was completed after 48-72 h of treatment with puromycin (4 μg/mL).

*Fluorescence microscope:* Cells grown on a coverslip or hierarchical hydrogels were fixed at room temperature with 4% paraformaldehyde for 30 minutes (min). Wash the fixed cells three times with 1×PBS for 5 min. Treating with 0.5% Triton X-100 at 4 °C for 10 min. Then, the cells were washed with 1×PBS containing 0.05% Tween-20 (1×PBS-T) three times, sealed with 10% BSA solution at room temperature for 1 h, stained with the required first antibody at 4 °C for 12-16 h, and then stained with the secondary antibody (1:300) for 1 h. For actin staining, the cells were incubated with Phalloidin-iFluor 647 reagent at 37 °C for 30 min. For nuclear staining, incubate the cells with DAPI/Hoechst 33342 for 15-20 min. Using 1×PBS-T, wash the cells three more times. Install the cover glass or hierarchical hydrogels on the confocal dishes, and use Leica SP8 laser confocal microscopy (Leica) for imaging. Use ImageJ or Leica software to post-process images. The following antibodies are used for immunostaining: Sec3 (1:200), Sec5 (1:200), and Miro1 (1:200).

*Scanning electron microscope (SEM):* Inoculate RAW 264.7 and BMSC together on a coverslip. After co-culturing for 24 h, the cell samples were fixed in 0.1M sodium bicarbonate buffer with 2.5% glutaraldehyde. The fixed sample was washed with sodium bicarbonate buffer and fixed with 0.1% OsO_4_. Perform gradient dehydration on the samples using 35%, 50%, 70%, 90%, and 100% ethanol solutions. Dry the samples that have been fixed and dehydrated, and place them on field emission scanning electron microscopy short columns for use with Au or Pt/Pd (5 nm). Imaging was performed on a field emission scanning electron microscopy (JEOL). Use ImageJ software to process images.

*Determination of ROS and TMRM:* BMSC (4×10^4^ cells per well) were inoculated into the hierarchical hydrogels. RAW 264.7 was treated with LPS for 24 hours before collecting cells for mitochondrial labeling using MitoTracker Deep Red. As with the previous treatment, after ensuring no excessive unbound MitoTrackers dye is inoculated onto the hierarchical hydrogels for co-culture. The incubated hierarchical hydrogel was incubated with MitoSOX Red (5 µM) or TMRM (100 nM). Then the hierarchical hydrogel was transferred to confocal dishes, the cells were imaged by confocal microscopy, and the mitochondrial ROS and TMRM levels of cells were analyzed by ImageJ software.

*Ultrastructural examination:* As mentioned, the harvested cells were fixed, dehydrated, permeabilized, and embedded in epoxy resin. To measure the TNT-mediated mitochondrial transfer between macrophages and BMSC and the characteristics of MDV nanogels-targeted macrophages, 70nm sections were observed by Talos 120kv frozen transmissionn electron microscopy (Thermo Fisher Scientific).

Immuno-TEM was used to identify mitochondria derived from macrophages in BMSC. We first transfected macrophages with Tom20-GFP lentivirus and then co-cultured them with BMSC. The treated cells were embedded in LR white resin. The grid was treated with 1% H_2_O_2_ in phosphate buffer solution, neutralized with ammonium chloride, sealed with 5% bovine serum albumin, incubated with primary antibodies against GFP (1:100), and then conjugated with 1.4nm nanogold. Use JEM 1400 transmission electron microscopy (JEOL) to check the samples at 120 kV and observe the positive points in BMSC.

*Detection of the inflammatory status of macrophages:* RAW 264.7 was treated with LPS and inoculated on hydrogel and MDI, respectively. The phenotype of macrophages was analyzed by flow cytometry. The collected cells were incubated with CD80-PE-Cy5 (1:200) antibodies at 4 °C for 30 min. Then, the proportion of CD80^+^ cells was analyzed using FACScan flow cytometry.

In addition, RAW 264.7 were divided into control, RAW+LPS+Gel, and RAW+LPS+Gel@MDI groups. The cultured cells were incubated with 10 µm DCFH-DA working solution at 37 °C for 20 min. Subsequently, the average fluorescence intensity was analyzed using flow cytometry (Beckmancoulter), and the data were analyzed using FlowJo software.

*In vitro osteogenesis properties:* Prepare mineralized cells for ALP staining after 7 days of cultivation. The cells were fixed with 4% PFA for 30 min and then stabilized with a BCIP/NBT alkaline phosphatase color development kit. After 14 days of induction, cells on the hierarchical hydrogels were stained with 0.1% Alizarin Red S solution (pH=4.6) for 5 min. Finally, thoroughly clean the cells with distilled water.

Detection of osteogenic marker gene expression. Obtain cDNA using total RNA extracted from BMSC cells. Perform RT-qPCR using the ABScript II One Step SYBR Green RT-qPCR Kit. *Gapdh* levels were normalized, and the relative expression levels were quantified using the 2-ΔΔCt method.

Primer sequences were as follows:

*Col1a* F: GCTCCTCTTAGGGGCCACT; R: CCACGTCTCACCATTGGGG

*Alp*: F: CCAACTCTTTTGTGCCAGAGA; R: GGCTACATTGGTGTTGAGCTTTT

*Runx2*: F: CCAACCGAGTCATTTAAGGCT; R: GCTCACGTCGCTCATCTTG

*Sp7*: F: ATGGCGTCCTCTCTGCTTG; R: TGAAAGGTCAGCGTATGGCTT

*Gapdh*: F: AGGTCGGTGTGAACGGATTTG; R: TGTAGACCATGTAGTTGAGGTCA

*Single-cell RNA Sequencing and data analysis:* The data on cranial defects were sourced from the Genome Sequence Archive (GSA) database under the accession number CRA005302. The sequencing data was initially preprocessed using the Cell Ranger pipeline (10× Genomics, Cellranger count v5) with default parameters and aligned to GRCh38 (v3.0.0). The resulting matrix files were used for downstream bioinformatics analysis.

Seurat (version 4.1.1) and R (version 4.1.2) were employed for further analysis. Cells with at least 500 detected genes and mitochondrial percentages less than 10% were retained. The data were then normalized to every 10,000 transcript copies and log-transformed to mitigate sequencing depth variation.

The UMAP method (RunUMAP, Seurat) was used to compute the manifold of 30 pre-calculated principal components for visualization and clustering. CellChat (1.6.0) was utilized to infer and analyze cell-to-cell communication. We employed netVisual_circle and netVisual_heatmap for the visualization of intercellular signal interactions.

To address the limitations of CellChat, we developed the cellPathwayHM function. This function visualizes all signals sent or received by a specific cell population and presents them as a heatmap. The source code for this function has been made publicly available on GitHub at the following address: https://github.com/GilbertHan1011/Macro_osteo_comm.

*Cell transfection and efficiency evaluation:* The Miro1 overexpression plasmid was constructed by Generay Technologies (Shanghai, China). Lipofectamine 3000, PEI, and Miro1 (MDV nanogels) were used for macrophage transfection, respectively. At the same time as cell inoculation, RAW 264.7 cells at a density of 10×10^4^ cells per well and 1000 µg/mL of MDV nanogels were inoculated into a 24-well plate together and co-cultured for 24 hours. Observe the transfection status of cells through confocal fluorescence photography 24 h after treatment. Perform data processing and analyze transfection efficiency through ImageJ software.

*RNA Sequencing and data analysis:* RNA from the sample was extracted using the RNAeasy Mini Kit according to the manufacturer's instructions. The quality and quantity of the extracted RNA were measured using a NanoDrop spectrophotometer (Thermo Fisher Scientific) and an Agilent 2100 Bioanalyzer (Agilent Technologies), respectively.

Library preparation and sequencing were performed at the XYZ Genomics Facility. Libraries were prepared using the TruSeq RNA Library Prep Kit (Illumina), following the manufacturer's protocols. Briefly, mRNA was purified from total RNA and fragmented. First-strand cDNA was synthesized using random hexamers and reverse transcriptase. Following the second strand synthesis, the cDNA was end-repaired, A-tailed, and ligated to Illumina sequencing adapters. The resulting libraries were size-selected, PCR amplified, and sequenced on an Illumina NovaSeq 6000, producing paired-end reads of 150 base pairs.

The Bcl2fastq software was used to perform base calling on the raw image data of sequencing results, and the pass filter data was obtained after preliminary quality analysis. The results were stored in FASTQ file format.

Raw reads were quality-checked with FastQC and trimmed of adapter sequences using Trimmomatic. The short reads were then aligned using the HISAT2 aligner. Gene expression was calculated using Htseq software. RPKMs at the current sequencing depth were evaluated by jackknifing using RSeQC, and the accuracy of the evaluated RPKM was measured by percent relative error.

Differential expression analysis was carried out utilizing the DESeq2 package in R, with a significance threshold set at an adjusted p-value of <0.05 and an absolute log2 fold change greater than 1. Gene enrichment was conducted via EnrichR (https://maayanlab.cloud/Enrichr/) and Gene Set Enrichment Analysis (GSEA). Data visualization, including dot plots and heatmaps, was rendered using ggplot2 and the ComplexHeatmap package, respectively. All the sequencing data are available from the Gene Expression Omnibus (GEO) under the accession code GSE239590.

*Micro-CT, histology, and immunofluorescence analysis:* Calvarial bone defect samples were subjected to overnight exposure to 4% paraformaldehyde and evaluated for *in vivo* bone formation using a micro-CT imaging system (Bruker). All fixed samples were scanned at a source of 20 kV tube voltage, 0.07 mA tube current, and 38 ms exposure time. A circular contour with a diameter of 3 mm was drawn around the center of each defect to determine the volume of interest. All data were analyzed using Imalytics Preclinical software, and the reconstruction parameters used to access new bone formation mainly composed of bone tissue volume/total tissue volume (BV/TV), trabecular thickness (Tb. Th), and trabecular separation (Tb. Sp). Then the sample was immersed in ethylenediaminetetraacetic acid (pH 7.2) for decalcification for 4 weeks and then embedded in paraffin. A paraffin section with a thickness of 4 μm was obtained and subjected to H&E staining and Masson’ Trichromic staining to qualitatively evaluate bone repair ability.

The sections were subjected to multiple fluorescence immunohistochemistry (TSA) for immunofluorescence staining. Primary antibodies with F4/80 (macrophage labeling) (1:100), iNOS (1:100), and CD206 (1:100). Stain the nucleus using DAPI. The slices were sealed with anti-fluorescence quenching sealant and scanned by a scanner KF-PRO-120/FL (Olympus, Japan). Three different regions of the same part were selected, and the corresponding fluorescence regions or positive staining cell numbers were semi-quantified using ImageJ software.

**References**

[1] Z. Zou, Z. Zhang, H. Ren, X. Cheng, X. Chen, C. He, *Biomaterials* **2023**, 301, 122251.


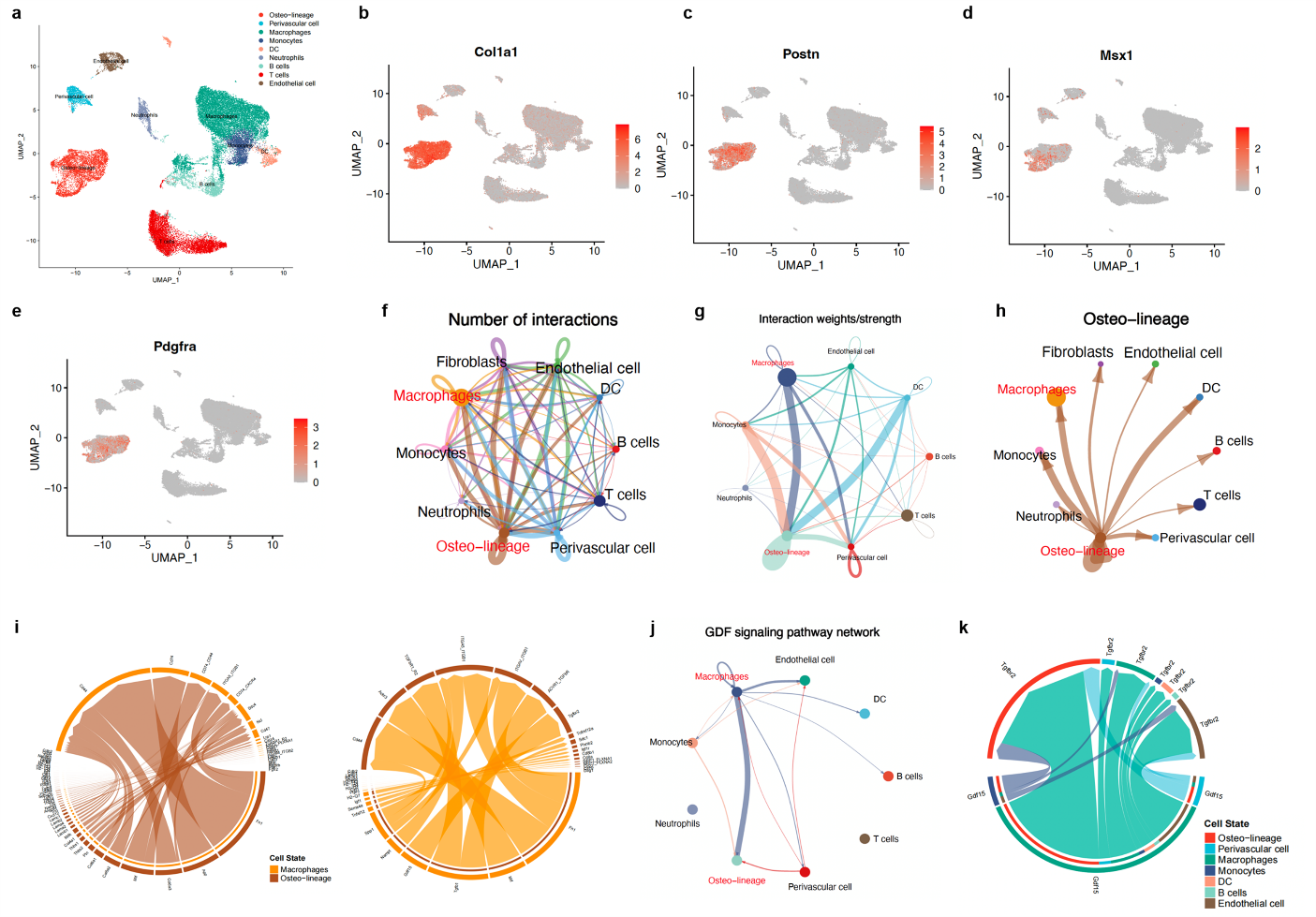


**Figure S1.** **Single-cell RNA sequencing (scRNA seq) of in situ cells during bone repair.** (a) A UMAP plot illustrates the distribution of 26,016 cells, categorized into 9 distinct sub-clusters. (b-e) These UMAP plots represent the expression of specific genes that characterize the osteo-lineage cell group. (f-g) Circle plots visually represent the interactions and their respective strengths among various cell populations. (h) This circle plot illustrates the magnitude of interactions initiated by the osteo-lineage group. (i) A chord diagram that portrays the interplay between the osteo-lineage and macrophage groups. The signals generated by the osteo-lineage group are presented on the left, while those emanating from the macrophage group are indicated on the right. (j) Circle plot presenting the interactions involving the GDF pathway among different cell populations. (k) A chord diagram illustrating all significant interactions (depicted as L-R pairs) associated with the GDF signaling pathways.

**
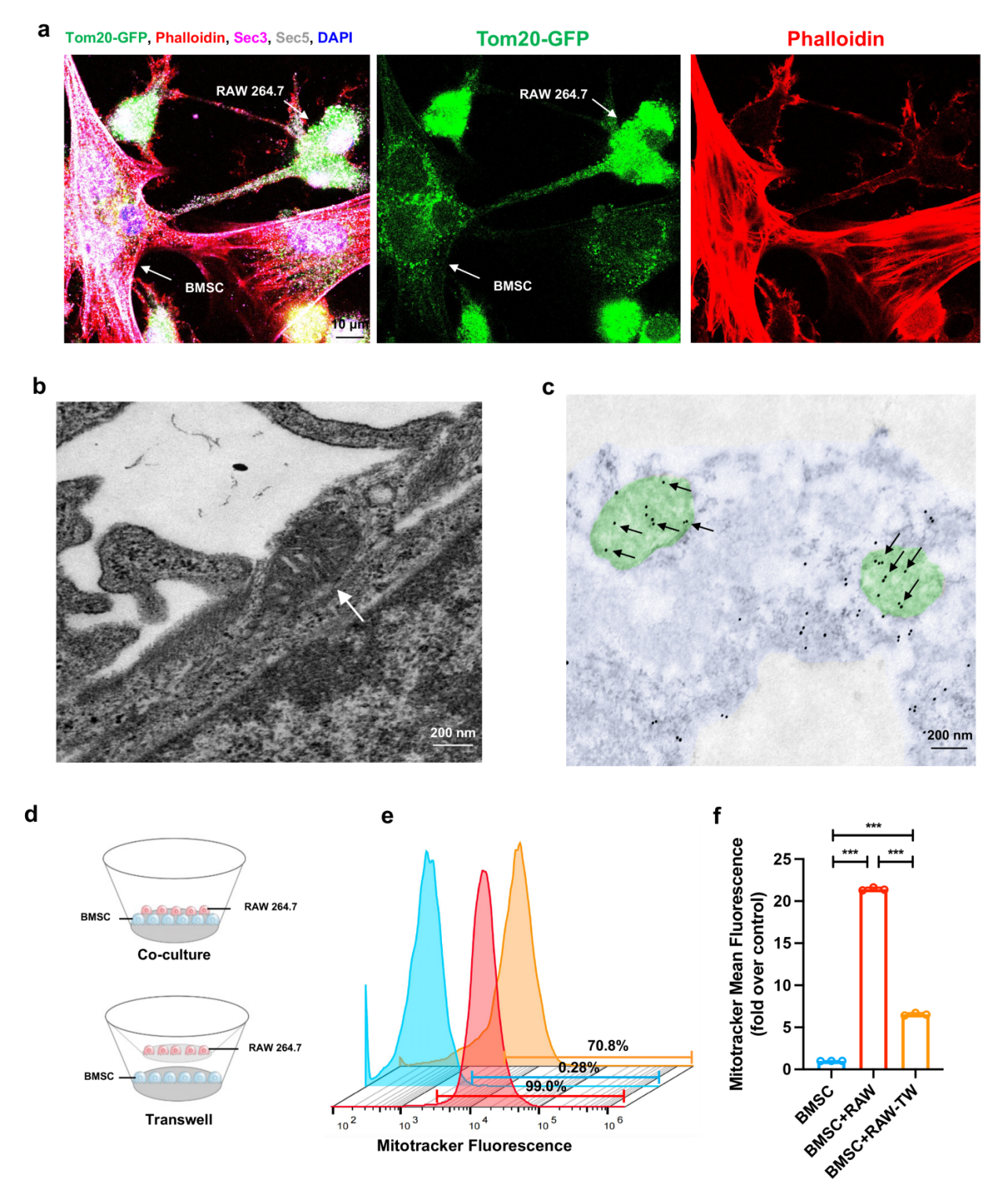
**

**Figure S2.** **Mitochondria transfer from RAW 264.7 to BMSC.** (a) Confocal image showing TNT-mediated transfer of Tom20-GFP-tagged mitochondria from macrophages to BMSCs. Mitochondria were labeled with Tom20-GFP. Actin was stained with phalloidin red. (b) Transmission electron micrographs (TEM) were taken after macrophage contact with BMSC. Macrophages transfer mitochondria by TNT. Scale bar, 200 nm. (c) Immuno-TEM images between the macrophage and the BMSC. Mitochondria are green. Unstained sections are pseudo‐colored. The comparison of GFP positive dots per cell. Scale bar, 200 nm. (d-f) The efficiency of mitochondrial transfer in the co-culture system and Transwell (TW) culture system (d). Flow cytometry analysis and average fluorescence intensity quantification of RAW264.7 and BMSC incubated in the TW culture system for 24 h. All statistical data are expressed as mean ± SD (n = 3; one‐way ANOVA with Tukey's post‐test. ****P* < 0.001.).

**
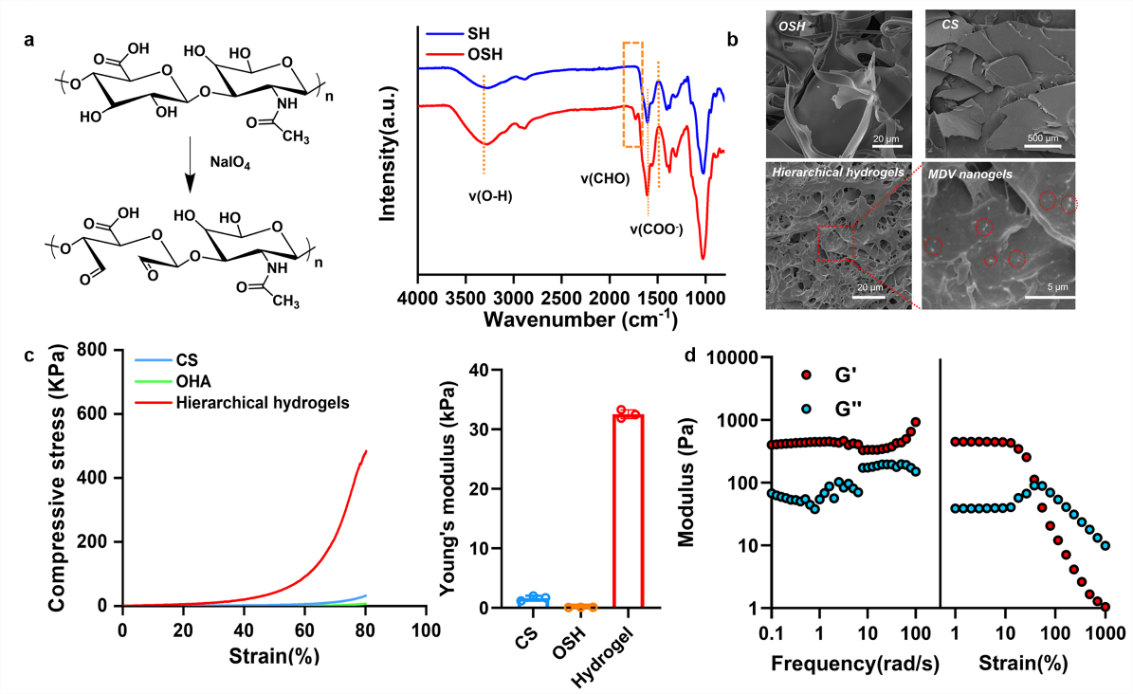
**

**Figure S3. The mechanical performance of hierarchical hydrogels.** (a) Synthesis route and FT-IR spectrum of OSH. (b) SEM images of OSH, CS, and hierarchical hydrogels with MDV nanogels. (c) Stress-strain curves of the OSH, CS, and hierarchical hydrogels by compressive measurements. (d) Oscillatory frequency sweep and strain sweep of hierarchical hydrogels.

**
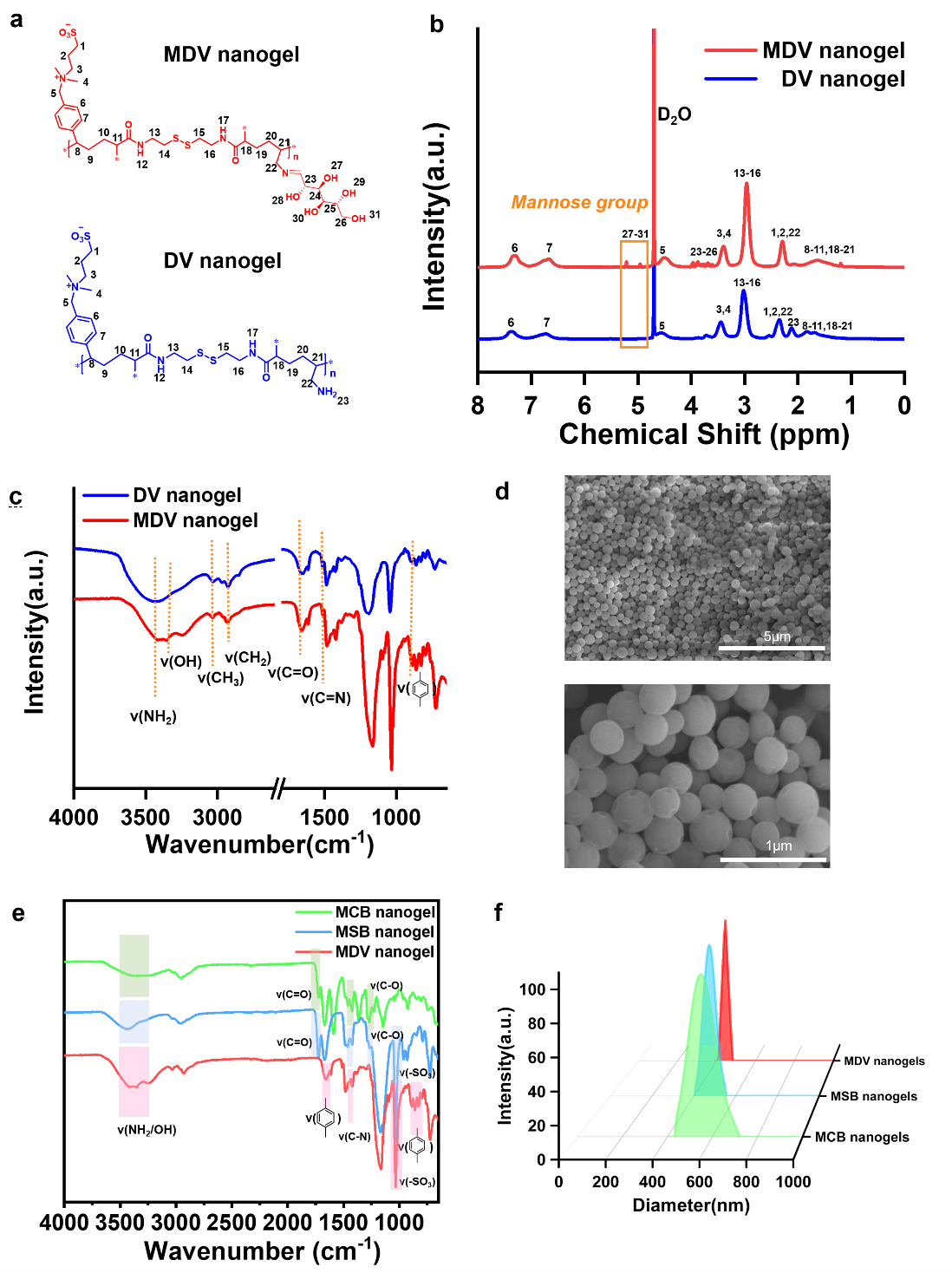
**

**Figure S4.** **The structure of zwitterionic nanogels.** (a, b) ^1^H-NMR (a) and FT-IR spectra (b) of DV nanogels and MDV nanogels. (c) SEM images of MDV nanogels with different magnifications. (e) The FT-IR spectra and (f) diameter of MCB, MSB, and MDV nanogels.

**
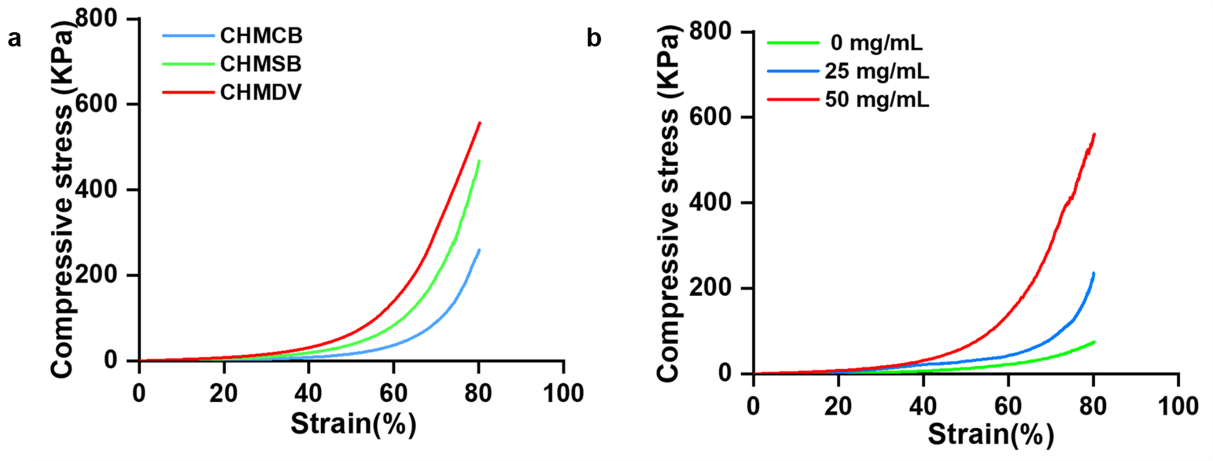
**

**Figure S5. Regulation of the optimal mechanobiological compatibility of hierarchical hydrogels for bone healing and regeneration.** (a, b) The compressive stress-strain curves of hierarchical hydrogels with (a) different zwitterionic nanogels (MCB, MSB, and MDV nanogels) and (b) different content of MDV nanogels (0, 25, and 50 mg/mL).

**
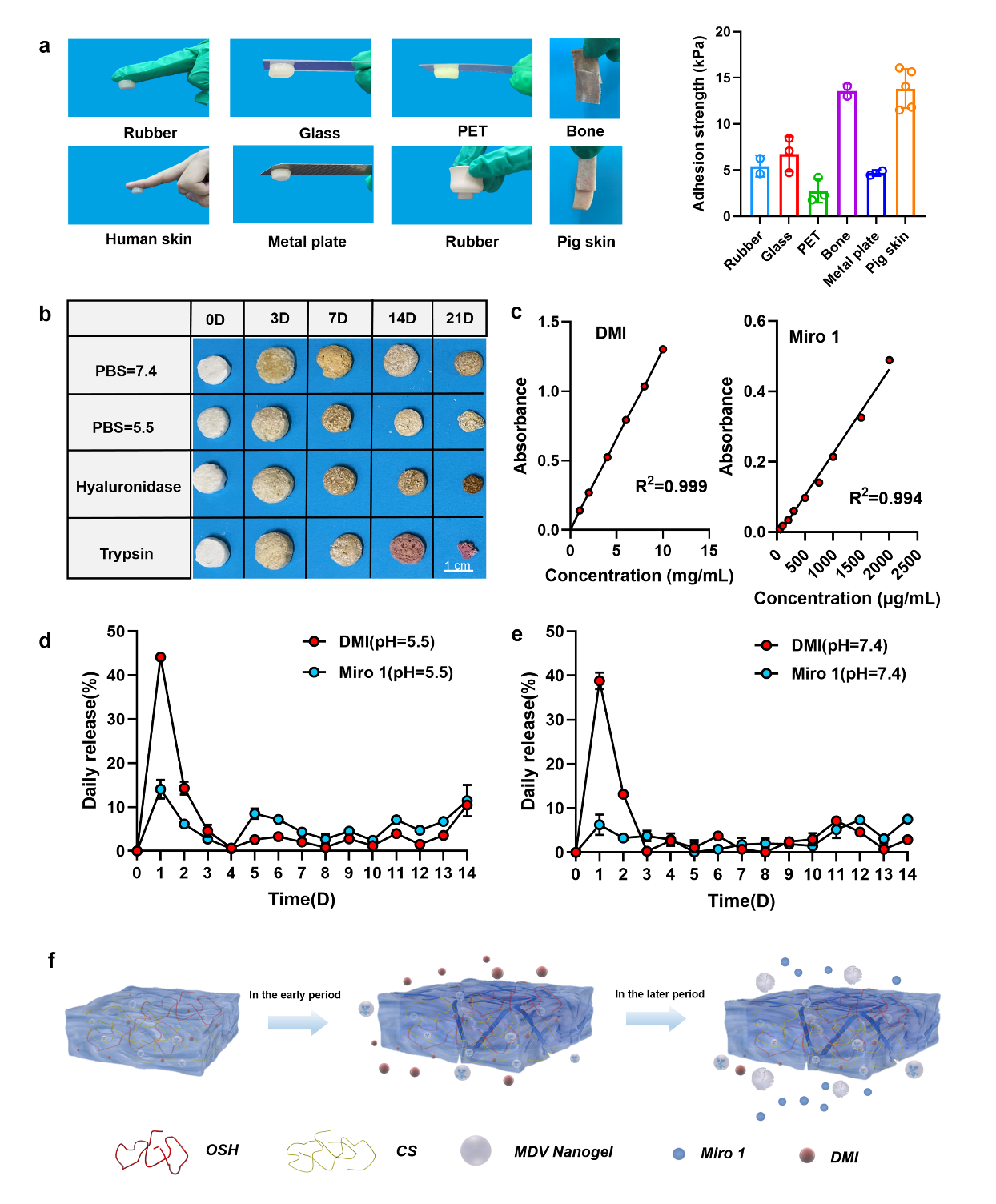
**

**Figure S6. Adhesion, degradation, and release performances of hierarchical hydrogels. (**a) Adhesion photograph and strength of hierarchical hydrogels to various substrates. (b) Degradation photographs of hierarchical hydrogels in different solutions, including phosphate-buffered saline (PBS) (pH=5.5 and 7.4), hyaluronidase, and trypsin. (c) Standard curves (absorbance-concentration) of DMI (λ=230 nm) and Miro 1 (λ=490 nm) were recorded by a microplate reader. (d, e) Daily release profiles of the DMI (d) and Miro 1 (e) from hierarchical hydrogels in different pH (5.5 and 7.4). (f) Schematic diagram for the hierarchical hydrogel releasing DMI and Miro 1.

**
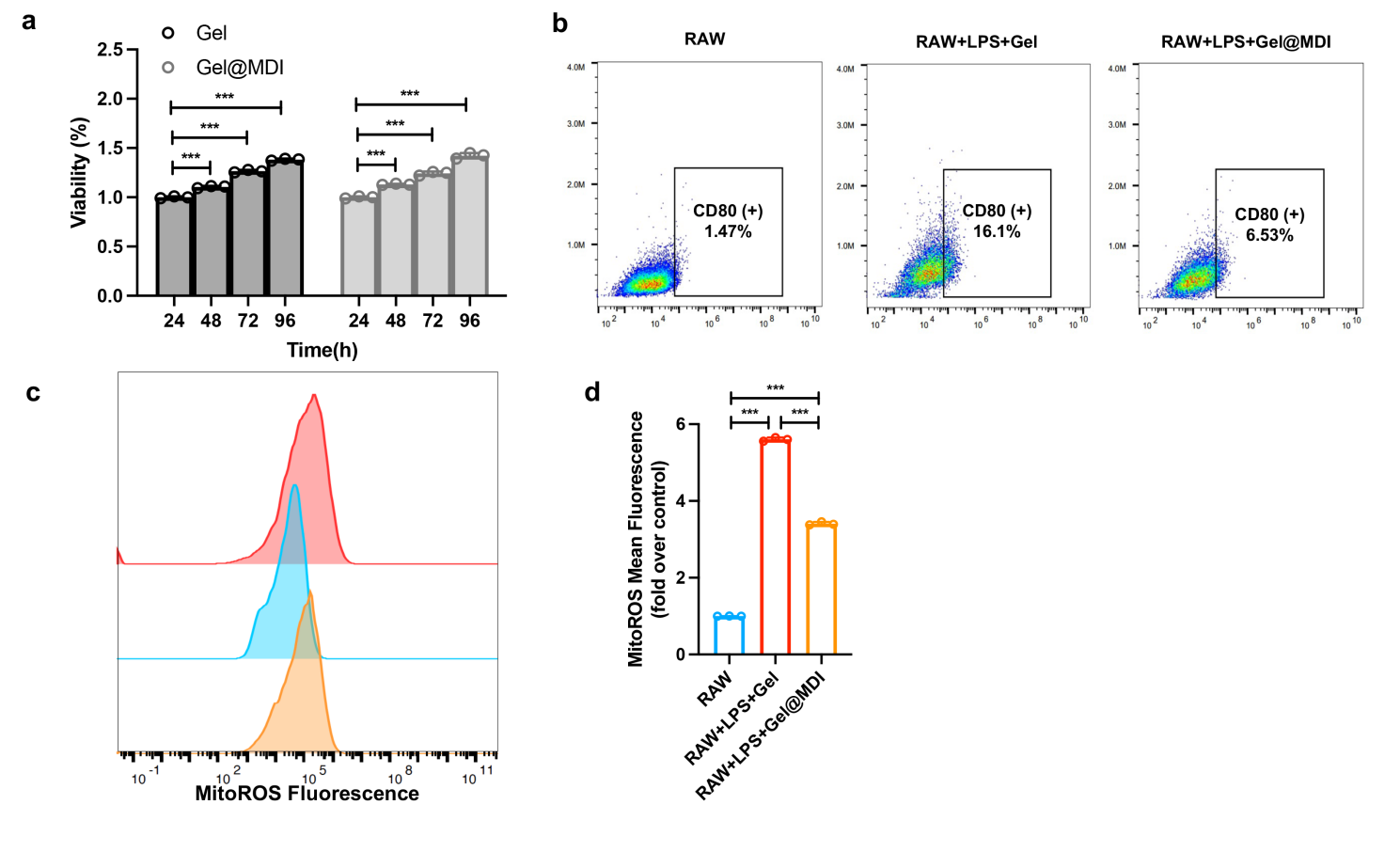
**

**Figure S7. Biocompatibility of the hierarchical hydrogels and phenotype identification of macrophage.** (a) CCK-8 analyzed that the hierarchical hydrogels were co-cultured with BMSC for 24 h (n = 3). (b) Quantitative analysis of CD80^+^ RAW 264.7 by flow cytometry. (c, d) Mitochondrial ROS production was analyzed by DCFH‐DA staining. The mean fluorescence intensity was quantified as a measure of ROS (n = 3). **P* < 0.05, ***P* < 0.01, ****P* < 0.001. (Note: Gel represented injected hydrogel framework, M represented Miro 1, D represented macrophage-targeted zwitterionic nanogels (MDV nanogels), and I represented anti-inflammatory drug dimethyl itaconate (DMI), respectively.)

**
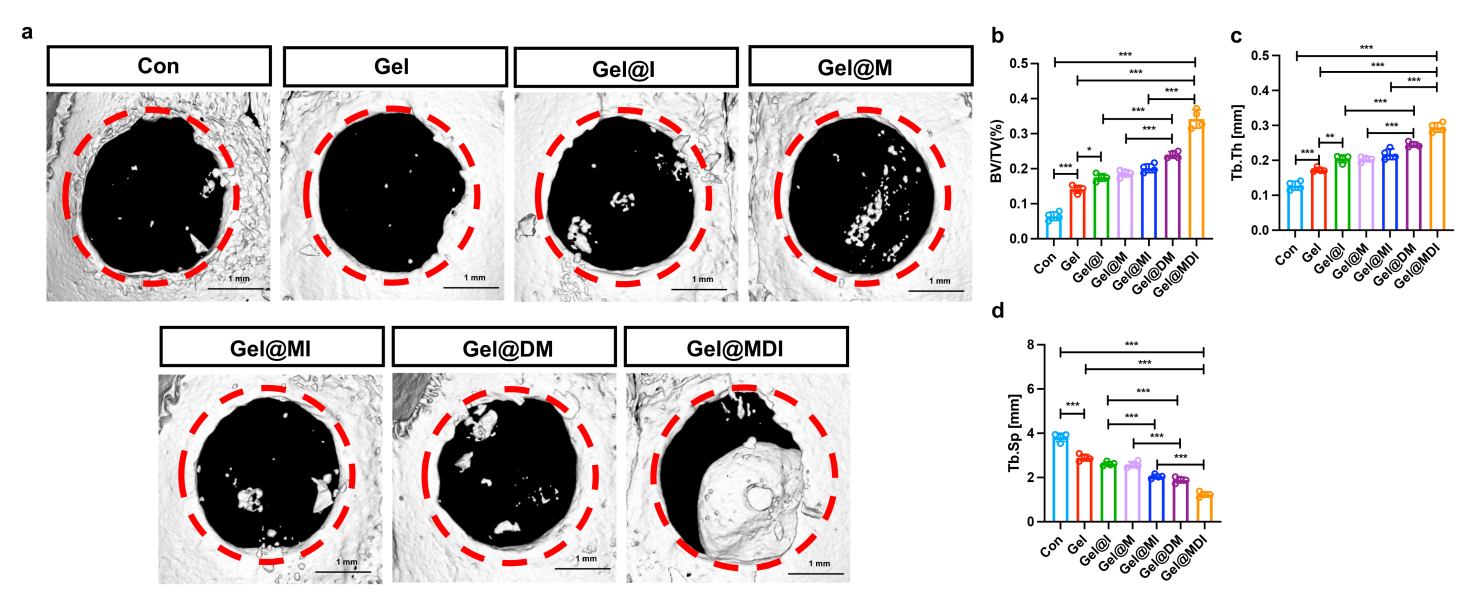
**

**Figure S8. *In vivo* evaluation of bone regeneration after treatment of calvarial defects with different hydrogels.** (a) Micro-CT images of calvarial defects implanted with different hydrogels. b-d) Quantitative analysis of newly regenerated bone tissues, including BV/TV (b), Tb.Th (c), and Tb.Sp (d) (n = 4). **P* < 0.05, ***P* < 0.01, ****P* < 0.001. (Note: Gel represented injected hydrogel framework, M represented Miro 1, D represented macrophage-targeted zwitterionic nanogels (MDV nanogels), and I represented anti-inflammatory drug dimethyl itaconate (DMI), respectively.)

**
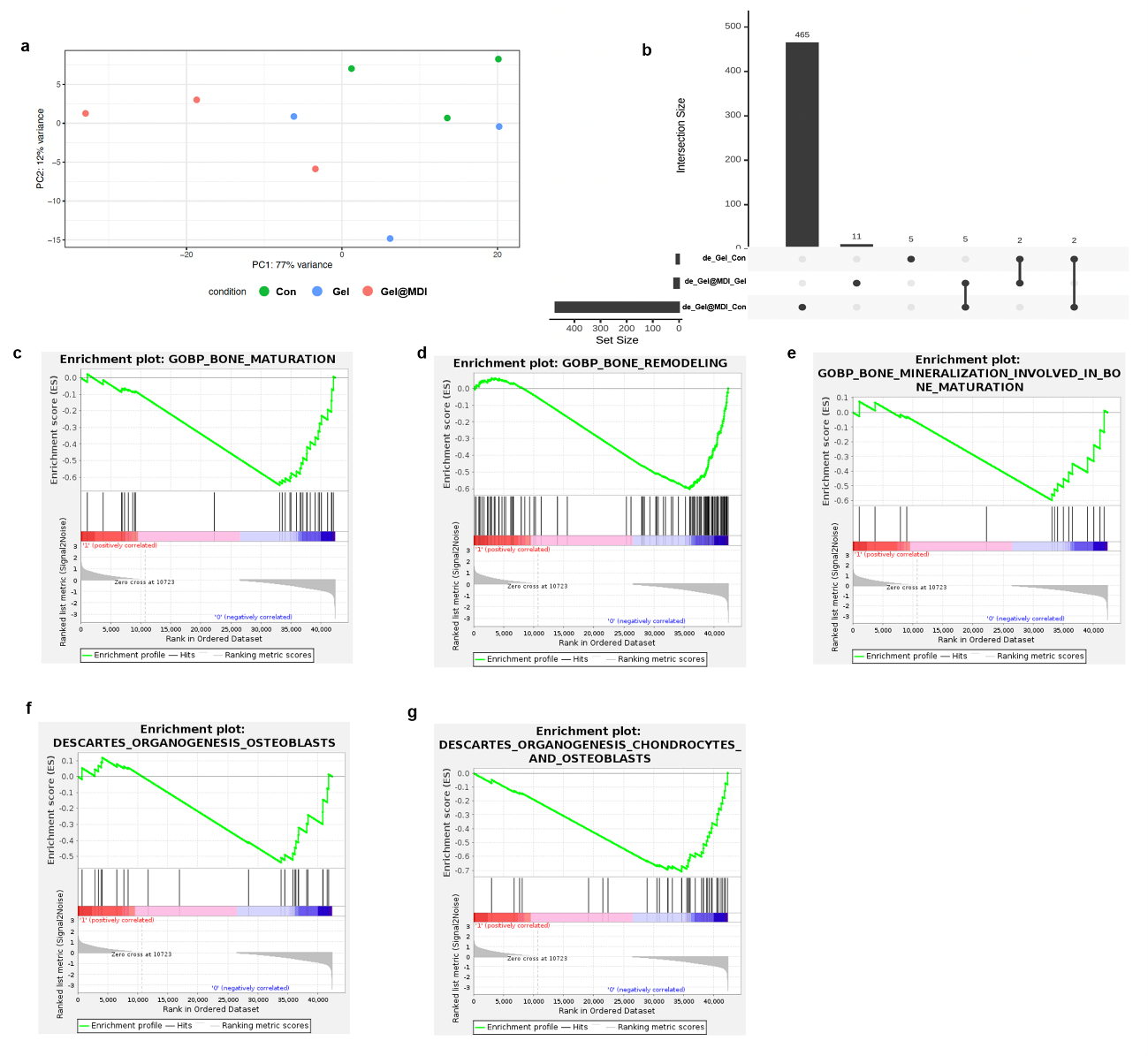
**

**Figure S9. Exploration of the osteogenic mechanism of the hierarchical hydrogels.** (a) Principle components analysis. (b) UpSet plot illustrates numbers of differentially expressed genes among three groups identified by DESeq2 analysis. (c-f) Gene set enrichment analysis.

**Table S1**. The composition of systems and abbreviation of related product.

| Abbreviation | the composition of systems | |
| --- | --- | --- |
| DMI | | dimethyl itaconate |
| Miro1 | | Rho GTPase 1 |
| CBMA | | Zwitterionic monomer |
| SBMA | | Zwitterionic monomer |
| DVBAPS | | Zwitterionic monomer |
| MCB | | mannose-modified CBMA nanogels |
| MSB | | mannose-modified SBMA nanogels |
| MDV | | mannose-modified MDV nanogels |
| CHMCB | | Hydrogel* with MCB nanogels |
| CHMSB | | Hydrogel with MSB nanogels |
| CHMDV | | Hydrogel with MDV nanogels |
| Gel (CH hydrogel) | | Hydrogel |
| Gel@I | | Hydrogel+DMI |
| Gel@M | | Hydrogel+ Miro1 |
| Gel@MI | | Hydrogel+ Miro1+DMI |
| Gel@MD | | Hydrogel+ Miro1+MDV nanogels |
| Gel@MDI | | Hydrogel+ Miro1+MDV nanogels+MDI |

*Note: Hydrogel consisted solely of chitosan and aldehyde-modified sodium hyaluronate.
